# The H-index of a network node and its relation to degree and coreness

**DOI:** 10.1038/ncomms10168

**Published:** 2016-01-12

**Authors:** Linyuan Lü, Tao Zhou, Qian-Ming Zhang, H. Eugene Stanley

**Affiliations:** 1 Alibaba Research Center for Complexity Sciences, Alibaba Business College, Hangzhou Normal University, Hangzhou 311121, China; 2 CompleX Lab, Web Sciences Center, University of Electronic Science and Technology of China, Chengdu 611731, China; 3 Big Data Research Center, Univesrsity of Electronic Science and Technology of China, Chengdu 611731, China; 4 Department of Physics and Center for Polymer Studies, Boston University, Boston, Massachusetts 02215, USA

## Abstract

Identifying influential nodes in dynamical processes is crucial in understanding network structure and function. Degree, H-index and coreness are widely used metrics, but previously treated as unrelated. Here we show their relation by constructing an operator 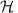, in terms of which degree, H-index and coreness are the initial, intermediate and steady states of the sequences, respectively. We obtain a family of H-indices that can be used to measure a node's importance. We also prove that the convergence to coreness can be guaranteed even under an asynchronous updating process, allowing a decentralized local method of calculating a node's coreness in large-scale evolving networks. Numerical analyses of the susceptible-infected-removed spreading dynamics on disparate real networks suggest that the H-index is a good tradeoff that in many cases can better quantify node influence than either degree or coreness.

## Abstract (web summary)

Identifying influential nodes in networks is important for the understanding of their structure and function, but there are several so far unrelated measures to assess this. Here, the authors unfold relations among knows criteria and construct a family of indices that interpolate between degree and coreness.

## Graphical abstract

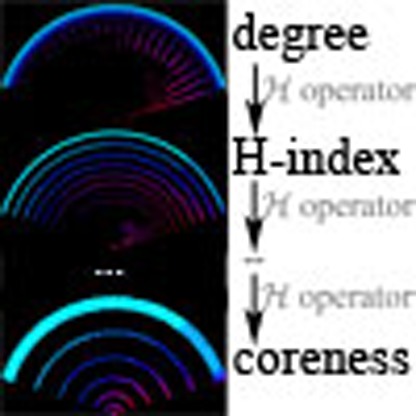

The focus of network science research has been shifting from discovering macroscopic statistical regularities^1^,^2^,^3^,^4^ to uncovering the role played by such microscopic elements as nodes, links and motifs in the structure and dynamics of the system^5^,^6^,^7^,^8^,^9^,^10^. Being able to effectively and efficiently identify the critical nodes associated with the specific dynamics of large-scale networks^11^ will allow us to better control the outbreak of epidemics^12^, conduct successful advertisements for e-commercial products^13^, prevent catastrophic outages in power grids or the Internet^14^, optimize the use of limited resources to facilitate information propagation^15^, discover drug target candidates and essential proteins^16^, and design strategies for communication breakdowns in human and telecommunication networks^17^.

The simplest way to measure the importance of a node is to determine its degree, that is, to count the number of its linked neighbours. Previous studies have shown that protecting, immunizing and regulating large-degree nodes can maintain network connectivity^18^, halt infectious disease propagation^12^, enhance synchronizability^19^, improve transport capacity^20^ and promote cooperation in evolutionary games^21^.

Recently, Kitsak *et al.*^22^ argued that the location of a node is more significant than the number of its linked neighbours, and they suggested that coreness is a better indicator of a node's influence on spreading dynamics than degree. The coreness of a node is measured by *k*-core decomposition^23^, and a larger coreness value indicates that the node is more centrally located in the network. The *k*-core decomposition process is initiated by removing all nodes with degree *k*=1. This causes new nodes with degree *k*≤1 to appear. These are also removed and the process is continued until the only nodes remaining are those of degree *k*>1. The removed nodes and their associated links form the 1-shell. This pruning process is repeated for the nodes of degree *k*=2 to extract the 2-shell, that is, in each stage the nodes with degree *k*≤2 are removed. The process is continued until all higher-layer shells have been identified and all network nodes have been removed. Then each node *i* is assigned a shell layer *c*_*i*_, called the coreness of node *i*. Recent studies suggest that coreness is a good measure of a node's influence^22^,^24^.

Calculating coreness requires global topological information, and its implementation is usually centralized, which can hinder its application to very large-scale dynamical networks. In contrast, degree is a simple local index, but of lower utility. As a tradeoff, Chen *et al.*^25^,^26^ proposed indices using the local neighbourhood information of individual nodes, which perform well but lack an underlying mathematical structure. This approach brings to mind the Hirsch index (also called the H-index)^27^, which was originally used to measure the citation impact of a scholar or a journal^27^,^28^,^29^. For a scholar or journal, the H-index is defined as the maximum value *h* such that there exists at least *h* papers, each with citation count ≥*h*.

Here we discuss the extension of the H-index concept to quantify how important a node is to its network^30^. The H-index of a node is defined to be the maximum value *h* such that there exists at least *h* neighbours of degree no less than *h*. In the Supplementary Fig. 1, we compare calculating the H-index of a scholar and the H-index of a node. Degree, H-index and coreness seem to be independent but are actually interrelated. We construct an operator 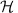 on a group of reals that returns a node's H-index when acting on its neighbours' degrees. By sequentially and synchronously applying the 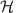 operator to each node, the returned value soon converges to coreness, that is, in terms of this operator, degree, H-index and coreness are its initial state, intermediate state and steady state, and all other intermediate states can also be treated as centrality measures. We further show that the convergence to coreness can be guaranteed even under an asynchronous updating process, thus allowing a distributed computing algorithm to deal with large-scale dynamical networks. To see whether these centralities can measure a node's influence, we apply the standard susceptible-infected-removed (SIR) spreading model^31^ on eight real networks from disparate fields and calculate the correlation between each node's influence and its centrality values. Simulation results show that the H-index outperforms both degree and coreness in several cases, and thus can be considered a good tradeoff between degree and coreness.

## Results

### Mathematical relationship

We construct an operator, 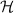, which acts on a finite number of reals (*x*_1_, *x*_2_, ⋯, *x*_*n*_) and returns an integer 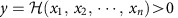, where *y* is the maximum integer such that there exist at least *y* elements in (*x*_1_, *x*_2_,⋯, *x*_*n*_), each of which is no less than *y*. For a scholar with *n* publications, *x*_1_, *x*_2_, ⋯, *x*_*n*_ is the number of citations to these publications and 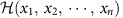 is the scholar's H-index.

Denote *G*(*V*, *E*) an undirected simple network, where *V* is the set of nodes and *E* is the set of links. The degree of an arbitrary node *i* is denoted by *k*_*i*_ and its neighbours' degrees are 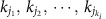. Then, we define the H-index of node *i*


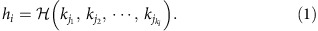


We define 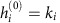 to be the zero-order H-index of node *i*, and define the *n*-order H-index (*n*>0) iteratively as


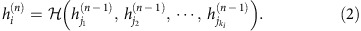


The H-index of node *i* is equal to the first-order H-index, namely 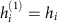. A more detailed illustration can be found in Supplementary Note 1.

*Theorem 1:* for every node *i*∈*V* of an undirected simple network *G*(*V*, *E*), its H-index sequence 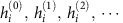 will converge to the coreness of node *i*,


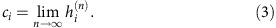


The proof is given in the Methods section. We give an example of iterative process from degree to coreness in the Supplementary Fig. 2.

This theorem shows that the degree, H-index and coreness are respectively the initial, intermediate and steady states under successive operations by 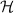. Given a network *G*(*V*, *E*), the convergence time *n*_∞_ is defined as the minimum number of iterations required to reach coreness from degree using the operator 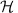, that is, *n*_∞_ is the minimum integer such that 
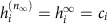
 for ∀*i*∈*V*.

Using equation (2) and Theorem 1, we implement the synchronous updating in eight representative real networks drawn from disparate fields, including two social networks (Sex and Facebook), two collaboration networks (Jazz and NS), one communication network (Email), one information network (PB), one transportation network (USAir) and one technological network (Router). In brief, Jazz^32^ is a collaboration network of jazz musicians and consists of 198 nodes and 2,742 interactions, NS^33^ is a co-authorship network of scientists working on network science, our Email^34^ network is of e-mail interchanges between members of the Rovira i Virgili University (Tarragona), our Sex^35^ network is of a bipartite sexual activity web community in which nodes are female (sex sellers) and male (sex buyers) and links between them are established when males write posts indicating sexual encounters with females, Facebook^36^ is a sample of the friendship network of Facebook users, PB^37^ is a network of US political blogs (the original links are directed, but here we treat them as undirected), USAir^38^ is the US air transportation network and Router^39^ is a symmetrized snapshot of the structure of the Internet at the level of autonomous systems. Their topological features are shown in Table 1. Experiments show that the sequence of H-indices quickly converges to the coreness (*n*_∞_ in Table 1). In addition to the degree, H-index and coreness, all other intermediate states *h*^(2)^, *h*^(3)^,⋯ can also be considered as centrality measures.

The resolution rate of the *h*^(*n*)^-index is the probability that two randomly chosen nodes will have different *h*^(*n*)^. It is also a useful index for measuring the degree to which a network is coarse grained. Note that degree is the most distinguishable index and coreness is the least distinguishable, and that the resolution rate decreases as the index order increases (Supplementary Fig. 3; Supplementary Note 2). On the other hand, the calculation of degree requires less information, while coreness requires the most information. For a given node *i*, the information required to calculate 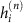 can be measured by information coverage, which is defined as the ratio of the number of nodes with a distance no more than *n* from *i* to the network size. The coverage rate increases as the index order increases (Supplementary Fig. 4). Surprisingly, we find that in some networks, such as NS and Router, the coverage rate is <1 even for 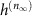, indicating that only partial information is required when calculating the coreness of a node in these networks (Supplementary Note 3).

**Figure 1:**
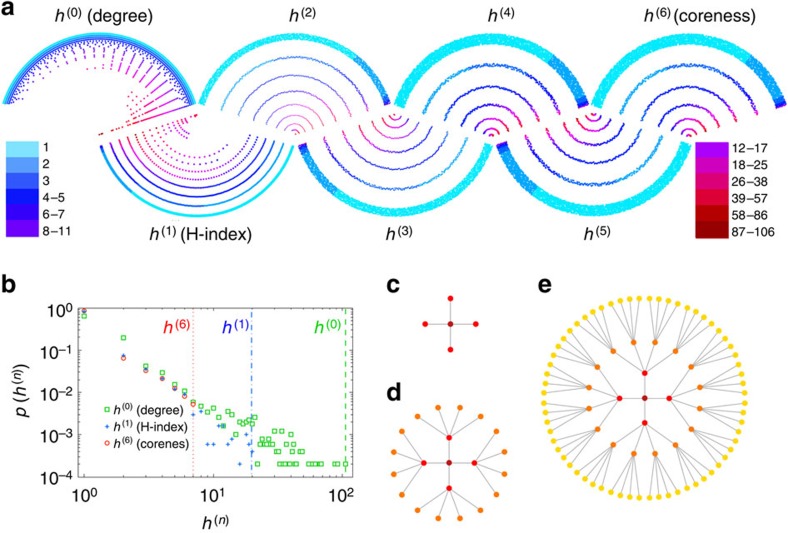
Comparisons among H-indices in different orders for the network Router. The subplot **a** exhibits a visualized illustration, where the colour represents the node degree (from 1 to 106). The node location represents the *h*^(*n*)^-index. Nodes located at a ‘fan' closer to the centre of the fan have higher *h*^(*n*)^ values, and nodes located at the same layer of a fan have the same *h*^(*n*)^ values. The subplot **b** shows the distributions of values of H-indices in different orders, where the green squares, blue crosses and red circles represent the cases for *n*=0, *n*=1 and *n*=6, respectively. The dash lines in different colours mark the largest values for the corresponding indices. The subplots **c**, **d** and **e** show an illustration of the hierarchical trees with 2, 3 and 4 levels, respectively.

Figure 1a shows the H-indices in different orders for a typical network Router. From left to right, we see the coarse graining process from degree to coreness. Figure 1b shows the probability distribution *p*(*h*), defined as the probability that a randomly selected node's *h*^(*n*)^ value is equal to *h* for the cases *n*=0, *n*=1 and *n*=*n*_∞_ of the network Router. Note that as the order *n* increases from 0 to 6 (*n*_∞_=6 for Router), the distribution narrows (see Supplementary Figs. 5–12 for the distributions of all H-family indices for the eight networks under study). Nevertheless, the distribution of values of high-order H-indices is still relatively broad, suggesting its potential as a good indicator of a node's importance^22^.

To show the different roles of different H-family indices, we iteratively construct a hierarchical tree (see ref. 40 for a similar method). As shown in Fig. 1c, the initialized network is of two levels, isomorphic to a star with *L* leaves. Here we set *L*=4. In each step, every leaf node becomes a star with *L* leaves, and the central node is connected with its original neighbours. After each step, the number of levels is increased by one, and the nodes in the more central positions (that is, of smaller levels) have a higher influence that is not reflected by their degree if the number of levels is high. Note that the three trees in Fig. 1c–e have 2, 3 and 4 levels, respectively, and in a hierarchical tree of *D* levels we need the index *h*^(0)^, *h*^(1)^, ⋯, *h*^(*D*−2)^ to quantify node influence. Such example clearly shows that a few low-order H-indices are not always enough to distinguish different nodes' influences.

### Asynchronous updating

The updating driven by 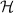 uses only local information, and it rapidly converges to coreness. However, the updating from *h*^(*n*−1)^ to *h*^(*n*)^ is implemented synchronously according to equation (2), and thus in principle requires a centralized controller to set up a global clock that records the order *n*. In particular, if the target network is evolving, the addition of a single link will require the recalculation of the entire sequence of H-indices. This limits the application of H-indices to large-scale dynamical networks. Fortunately, the asynchronous updating can still guarantee a convergence to coreness, as shown in the following theorem.

*Theorem 2:* given an undirected simple network *G*(*V*, *E*), for every node *j*∈*V*, we define *g*_*j*_=*k*_*j*_. In each iteration of the asynchronous updating process, a node *i* is randomly selected and its *g* value updated, that is,


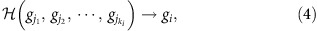


where 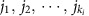 are the neighbouring nodes of *i*. If 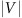 is finite, this updating process will reach a steady state 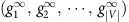 after a finite number of iterations such that the updating at any node will not change its *g* value, namely,


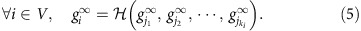


In the steady state, for every node *i* we have 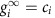. The proof is given in the Methods section.

Note that in the updating process of equation (4) the *g* values are not associated with a temporal superscript. In fact, at a certain updating step the values 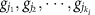 could lie in different stages, some updated dozens of times and others never updated. Thus, for any node *i*, before it reaches the steady state 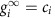, all the intermediate values are not necessarily equal to any order of H-indices. Theorem 2 makes a significant step towards making feasible applications to large-scale dynamical networks possible: it guarantees that a decentralized and localized method can be used to calculate the coreness, and even if the network evolves in time, equation (4) can be used to calculate the coreness when new links and nodes are periodically added, and all the previously obtained *g* values are still usable.

### Quantifying spreading influences

Epidemic spreading, one of the most significant dynamic behaviours in complex networks^41^, can be used to characterize such real processes as the spreading of infectious diseases^42^, the diffusion of microfinance^43^ and the propagation of traffic congestion^44^. To see whether the H-indices can quantify the spreading of node influence, we study the standard SIR spreading model^41^ in which the influence *R*_*i*_ of node *i* is quantified using the average number of removed nodes after the dynamics over 1,000 independent runs, each of which begins with node *i* as the sole infected seed (see details in Methods).

Given the order *n* (0≤*n*≤*n*_∞_), we have two sequences associated with the 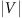 nodes: the *h*^(*n*)^-index 
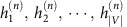
 and the influences 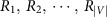. To quantify to what extent the *h*^(*n*)^-index resembles node influence values, we apply the Kendall Tau (*τ*) coefficient^45^, which lies in the range −1≤*τ*≤1. The larger value of *τ* means a stronger correlation between the two sequences (see Methods for the definition of *τ*).

**Figure 2:**
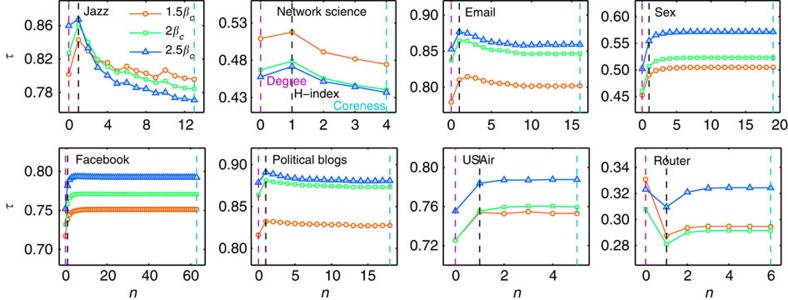
The Kendall Tau between *h*^(*n*)^-index and the node influence index *R* for undirected networks. The value of *n* ranges from 0 to *n*_∞_. The red circles, green squares and blue triangles represent the case of *β*=1.5*β*_*c*_, 2*β*_*c*_ and 2.5*β*_*c*_, respectively. The dash lines in purple, black and cyan colours emphasize the *τ* values for degree (that is, *h*^(0)^), H-index (that is, *h*^(1)^) and coreness (that is, *h*^∞^), respectively. The influence *R* of a node is quantified using the average number of removed nodes after the dynamics over 1,000 independent runs.

Figure 2 shows that the H-index of node *i* is highly correlated with the influence value *R*_*i*_. In many cases (Jazz, NS, Email and PB), the H-index (that is, *h*^(1)^-index) outperforms both degree and coreness. In other cases (Sex, USAir and Facebook), coreness outperforms degree and the performance of low-order H-indices rapidly approaches that of coreness as the order increases from zero. Router is an exception, where degree performs the best and the H-index the worst. Note that because Router is the most sparse network it may hinder spreading and make predicting influences more difficult (as indicated by the smallest *τ* value). Thus, the sequence of H-indices, starting from degree and driven by the operator 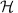, provides more alternative centralities in characterizing the importance of nodes, and the low-order H-indices are a good tradeoff between degree and coreness. We further compare the three best known H-indices (degree, H-index and coreness) with two well-known centrality indices (closeness and betweenness) for undirected networks. The definitions of closeness and betweenness are given in Methods. As shown in Table 2, all the H-family indices are competitive, and the H-index and closeness are the overall best performers, but the computational cost in calculating closeness is huge and thus the H-index is the better choice.

We further test two well-known dynamical processes: the susceptible-infected-susceptible (SIS) spreading model^46^ and bond percolation^47^. In the SIS model, the node influence index *R* is defined as the probability that this node will remain infected in the steady state. In bond percolation, the node influence index *R* is defined as either the probability that the target node belongs to the giant component or the size of the connected component that encompasses the target node. The results (Supplementary Tables 3–11) suggest that the H-family indices are competitive, especially for the H-index and coreness. Detailed information about the dynamical processes and the simulation results can be found in Supplementary Note 4.

## Discussion

We discover an important relation among degree, H-index and coreness—centrality measures that have previously been treated as unrelated. We construct an operator 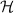 that functions as a ‘necklace' stringing together degree, H-index, coreness and other intermediate indices. All these indices are centralities that characterize each node's importance. Using the operator 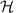 to achieve the coreness looks like an inverse way to the iterative removal of nodes with degree less than *k* that is widely used to determine the *k*-core of a network. Indeed, they are different, as the iterative removal method cannot result in H-index, or any other H-family indices except for degree and coreness, and the steps required to achieve the final coreness for the two methods are also different.

Although the importance of a given node strongly depends on the type of dynamical processes under consideration and thus there is no single best centrality measure, we need effective and elegant centralities in practice. For example, although we know that degree is not an accurate centrality measure in quantifying node influence in many dynamical processes^9^,^22^, it is still a useful estimation of node importance, even without the specification of dynamics. In despite of its bias and disadvantages^48^,^49^,^50^ the H-index is now becoming the most widely applied index for academic performances, ranging from individual scientists, scientific journals to universities and even countries. As indicated by the simulations on the SIR model, the SIS model and bond percolation, the H-family indices are effective in quantifying the spreading influences of nodes.

The asynchronous updating by 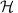 can still guarantee the convergence to coreness, and thus one can use a decentralized local algorithm to calculate coreness, which is able to deal with evolving networks. However, randomly selecting nodes to update in each iteration may greatly extend the time required before arriving at the steady state, even in static networks. Thus, the process for selecting which node to update is a nontrivial issue. For example, we can shorten the convergence time by reducing the selection probability of nodes that have been updated many times but have *g* values seldom changed. In addition, the change of *g* value of a node will enhance the updating probabilities of its neighbouring nodes, making this issue more complicated and thus more interesting.

The methodologies and results presented here are also applicable to directed networks and weighted networks, in which degree is replaced by in-degree, out-degree or node strength. In this way we can also define the H_in_-index, H_out_-index and weighted H-index, as well as in-coreness, out-coreness and weighted coreness. An example of how to calculate the directed H-family indices is presented in Supplementary Fig. 13. To test the performance, we compare the directed H-family indices with PageRank^51^ and HITs^52^ on seven directed networks. The basic statistics of these seven directed networks are summarized in Supplementary Table 12 and a more detailed introduction given in Supplementary Note 5. The results (Supplementary Tables 13–24) suggest that the directed H-family indices are still very competitive (in-coreness performs overall best).

## Methods

### Proof of Theorem 1

From the definition of 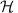-function, for any node *i* and integer *n*≥0, we have 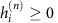 and 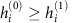. Applying mathematical induction, we prove that 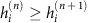 for any node *i* and integer *n*≥0. If 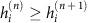 is valid for all *n*≤*m*, we then prove it to also be valid for *n*=*m*+1. From the definition, 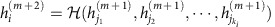, according to the induction hypothesis, 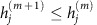 for any node *j*. Therefore, 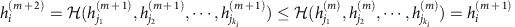, namely 
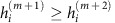
. Since 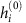, 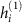, 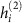, ⋯ is a monotonously nonincreasing sequence, and each element is nonnegative, it has a nonnegative limitation. Thus, we can define 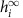 as the limitation of the sequence 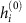, 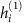, 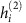, ⋯.

We then introduce two simple relations. First, if *G*′(*V*′, *E*′)⊆*G*(*V*, *E*), then from the definition of an 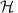-function it is obvious that for any node *i*∈*V*′ and any integer *n*≥0, 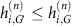, where the subscript *G*′ indicates that the corresponding index is defined on the subgraph *G*′. Second, if we denote *k*_min_ the minimal degree of *G*, then for any node *i* and any integer *n*≥0, 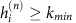. This second relation can also be proven by mathematical induction. It is clear that it is valid for *n*=0. We next prove that if this relation is valid for all *n*≤*m*, then it is also valid for *n*=*m*+1. For any node *i*, 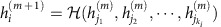, and according to the induction hypothesis, all elements in 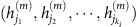 are no less than *k*_min_ and the number of elements in 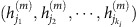 is *k*_*i*_≥*k*_min_. Therefore, according to the definition of 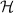-function, 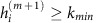.

If we denote *G*′ the *c*_*i*_-core of *G*, it is clear that *G*′⊆*G* and in *G*′, *k*_min_≥*c*_*i*_. Therefore, 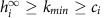. We denote *G*″(*V*″, *E*″) the induced subgraph containing all nodes *j* such that 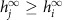. Note that the node *i* itself also belongs to *G*″. For any node *l*∈*V*, we find 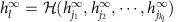, where 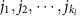 are the *k*_*l*_ neighbours of node *l*. For any node *j*∈*V*″, since in *G*, 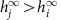, there are at least 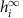 neighbours of node *j* with *h*^∞^ values no less than 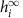. Thus, these neighbours also belong to *V*″. Therefore, in *G*″ the degrees of all the nodes are no less then 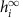, that is, *G*″ is a subgraph of *G*'s 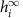-core. Because *c*_*i*_ is the coreness of *i*, 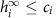. Combining the two inequalities, we arrive at Theorem 1.

### Proof of Theorem 2

For convenience, we introduce the systematic time step *t*. Initially we set *t*=0, and for every node *j*∈*V* we define 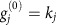. Then, at each time step, we randomly select a node and perform the 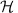 operator on it. If at time step *t*>0 the node *i* is selected, then 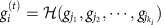. The *g* value without a temporal superscript indicates the most recently updated value, since only the current value is meaningful in the asynchronous updating procedure. Note that all neglected superscripts are smaller than *t*. Note also that for an arbitrary pair (*t*, *j*), 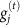 may not exist unless the node *j* is selected at time step *t*.

We first prove that if any node *j*∈*V* has been selected at time steps *t*_1_ and *t*_2_, and *t*_2_>*t*_1_≥0, then 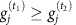. Note that for any node *j* selected at time step *t*=1 we have 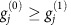. We apply mathematical induction and assume that the above inequality holds when *t*_1_≤*n* and *t*_2_≤*n*, and we next prove this also holds for *t*_1_≤*n*+1 and *t*_2_≤*n*+1. If node *i* is the one selected at time step *t*=*n*+1, then the aforementioned inequality holds for all other nodes *j≠i*. We denote *t*′ (0≤*t*′≤*n*) an arbitrary earlier updating time step of node *i*, and record two updates, that is, 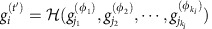 and 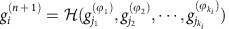. Note that for any *m* (1≤*m*≤*k*_*i*_) we have *φ*_*m*_≤*ϕ*_*m*_≤*n*. According to the induction hypothesis, 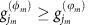, together with the definition of 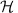 function, we have 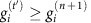. We denote the updating time steps of any node *i*∈*V* to be 0=*t*_0_<*t*_1_<*t*_2_< ⋯, then 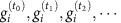 is a monotonously nonincreasing sequence and each element is nonnegative, and therefore it has a nonnegative limitation. At this point, we can define 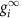 as the limitation of the sequence 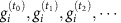.

We first prove that for any node *j*∈*V*, 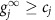. Proving by contradiction, when this inequality does not hold we denote *i* to be the first node to reach a *g* value smaller than *c*_*i*_, and the corresponding updating time step is *t*, that is, 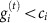 and before *t* for all nodes *j*∈*V*, *g*_*j*_≥*c*_*j*_. Note that *g*_*j*_ without a superscript indicates the last updated value before *t*. Therefore, 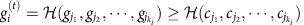. According to Theorem 1, 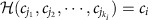, namely 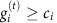. This leads to a contradiction and thus the inequality 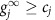 is validated.

Analogous to the proof of Theorem 1, after convergence, for any node *i*∈*V*, all nodes *j* such that 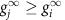, including *i* itself, constitute an induced subgraph of *G*'s 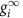-core. Since *c*_*i*_ is the coreness of *i*, 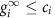. Combining the two inequalities, we arrive at Theorem 2.

### Spreading models

The standard SIR model, also referred to as the susceptible-infected-recovered model, is usually applied to analyse the propagation of opinions or news^41^. In the SIR model, there is a group of infected seed nodes and all other nodes are initially susceptible. At each time step, each infected node makes contact with its neighbours and each susceptible neighbour is infected with a probability *β*. Then, each infected node enters the removed state with a probability *λ*. For simplicity, we set *λ*=1. To quantify the spreading influence of a target node *i*, we begin the spreading process with *i* being the sole infected seed. When there are no longer any infected nodes and the dynamic process ends, the number of removed nodes *R*_*i*_ is a measurement of the influence of node *i*. Because we use small *β* values in our simulations, the infected percentage of the population is also small. When *β* values are high the disease infects a large percentage of the population, irrespective of where it originated, and the influence of individual nodes cannot be measured. According to the heterogeneous mean-field theory^47^,^53^,^54^, the epidemic threshold of SIR model is approximate to 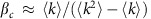. To be more precise, we determine the epidemic threshold *β*_*c*_ by simulation on real networks^55^. We set *β*=1.5*β*_*c*_, 2*β*_*c*_ and 2.5*β*_*c*_ in this paper, and we have checked that the choice of theoretical or simulation threshold will not affect the conclusion. Because the fluctuation of *R*_*i*_ is large when *β* values are small, we use 1,000 independent implementations for averaging.

### Benchmark centralities

We also compare two benchmark centrality indices (that is, closeness and betweenness) for undirected networks. Betweenness is one of the most popular geodesic-path-based ranking measures. It is defined as the fraction of shortest paths between all node pairs that pass through the node of interest. Betweenness is, in some sense, a measure of the influence of a node in terms of its role in spreading information^20^,^56^. For a network *G*=(*V*, *E*), the betweenness centrality of node *i*, denoted by *B*(*i*), is defined as^57^,^58^


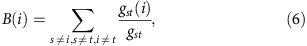


where *g*_*st*_ is the number of shortest paths between nodes *s* and *t*, and *g*_*st*_(*i*) denotes the number of shortest paths between nodes *s* and *t* that pass through node *i*.

Closeness of node *i* is defined as the reciprocal of the sum of geodesic distances to all other nodes of *V*^59^,^60^,


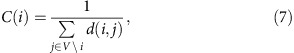


where *d*(*i*, *j*) is the geodesic distance between *i* and *j*. Closeness can be used to measure of how far information will be able to spread from a given node to other reachable nodes in the network.

### The Kendall Tau

We consider two sequences associated with 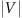 nodes, 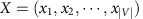 and 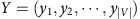, as well as the 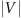 two-tuples 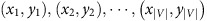. Any pair of two-tuples (*x*_*i*_, *y*_*i*_) and (*x*_*j*_, *y*_*j*_) (*i* ≠ *j*) are concordant if the ranks for both elements agree, that is, if both *x*_*i*_>*x*_*j*_ and *y*_*i*_>*y*_*j*_ or if both *x*_*i*_<*x*_*j*_ and *y*_*i*_<*y*_*j*_. They are discordant if *x*_*i*_>*x*_*j*_ and *y*_*i*_<*y*_*j*_ or if *x*_*i*_<*x*_*j*_ and *y*_*i*_>*y*_*j*_. If *x*_*i*_=*x*_*j*_ or *y*_*i*_=*y*_*j*_, the pair is neither concordant nor discordant. Comparing all 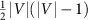 pairs of two-tuples, the Kendall Tau is defined as 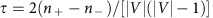, where *n*_+_ and *n*_−_ are the number of concordant and discordant pairs, respectively. The coefficient must be in the range −1≤*τ*≤1, and if *X* and *Y* are independent, *τ* should be approximately zero and thus the extent to which *τ* exceeds zero indicates the strength of the correlation.

### Notations

We summarize the notations used in this paper in Supplementary Table 25.

## Additional information

**How to cite this article:** Lü, L. *et al.* The H-index of a network node and its relation to degree and coreness. *Nat. Commun.* 7:10168 doi: 10.1038/ncomms10168 (2016).

## Supplementary Material

Supplementary Information Supplementary Figures 1-13, Supplementary Tables 1-25, Supplementary Notes 1-5 and Supplementary References.

## Figures and Tables

**Table 1:** The basic topological features and the convergence time of the eight real networks.

**Networks**	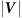	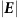	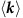	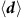	***C***	***r***	***n***_**∞**_
Jazz	198	2,742	27.7	2.24	0.633	0.02	13
NS	379	914	4.82	6.04	0.798	−0.082	4
Email	1,133	5,451	9.62	3.61	0.254	0.078	16
Sex	15,810	38,540	4.88	5.79	0	−0.115	19
Facebook	63,731	817,090	25.64	8.09	0.253	0.177	63
PB	1,222	16,714	27.36	2.74	0.360	−0.221	18
USAir	332	2,126	12.81	2.74	0.749	−0.208	5
Router	5,022	6,258	2.49	6.45	0.033	−0.138	6

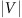
 and 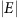 are the number of nodes and links, respectively. 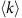 and 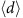 are the average degree and the average distance, respectively. *C* and *r* are the clustering coefficient^1^ and assortative coefficient^3^, respectively. Nodes with degree 1 are excluded from the calculation of clustering coefficient. Sex is a bipartite network and thus is characterized by (or ‘has') clustering coefficient zero. *n*_∞_ is the convergence time to coreness, defined as the minimum steps required to reach coreness from degree by the operator 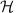.

**Table 2:** The Kendall Tau between the node influence index *R* of SIR model and five centrality indices.

**Networks**	**Degree**	**H-index**	**Coreness**	**Closeness**	**Betweenness**
Jazz	0.8021	**0.8431**	0.7958	0.6961	0.4629
NS	0.5092	**0.5178**	0.4747	0.3510	0.3392
Email	0.7794	**0.8103**	0.8021	0.7747	0.6195
Sex	0.4525	0.4905	0.5049	**0.7029**	0.3834
Facebook	0.7173	0.7381	**0.7513**	0.6716	0.4851
PB	0.8159	**0.8321**	0.8274	0.7375	0.6589
USAir	0.7256	**0.7540**	0.7529	0.7453	0.5442
Router	0.3309	0.2877	0.2946	**0.5975**	0.3228

SIR, susceptible-infected-removed.

They are degree (that is, *h*^(0)^), H-index (that is, *h*^(1)^), coreness (that is, *h*^∞^), closeness and betweenness. The spreading rate *β* is set as *β*=1.5*β*_*c*_, and for other values of *β*, the results are very similar and can be found in the Supplementary Table 1 (*β*=2*β*_*c*_) and Supplementary Table 2 (*β*=2.5*β*_*c*_). In each row, the largest *τ* is highlighted in bold.
