# Supplementary material for: The H-index of a network node and its relation to degree and coreness
Source: Nat Commun. 2016 Jan 12;7:10168. doi: 10.1038/ncomms10168 (PMC4729922; doi:10.1038/ncomms10168)
Supplement: Supplementary Information — Supplementary Figures 1-13, Supplementary Tables 1-25, Supplementary Notes 1-5 and Supplementary References. [file ncomms10168-s1.pdf]

# Supplementary Figures

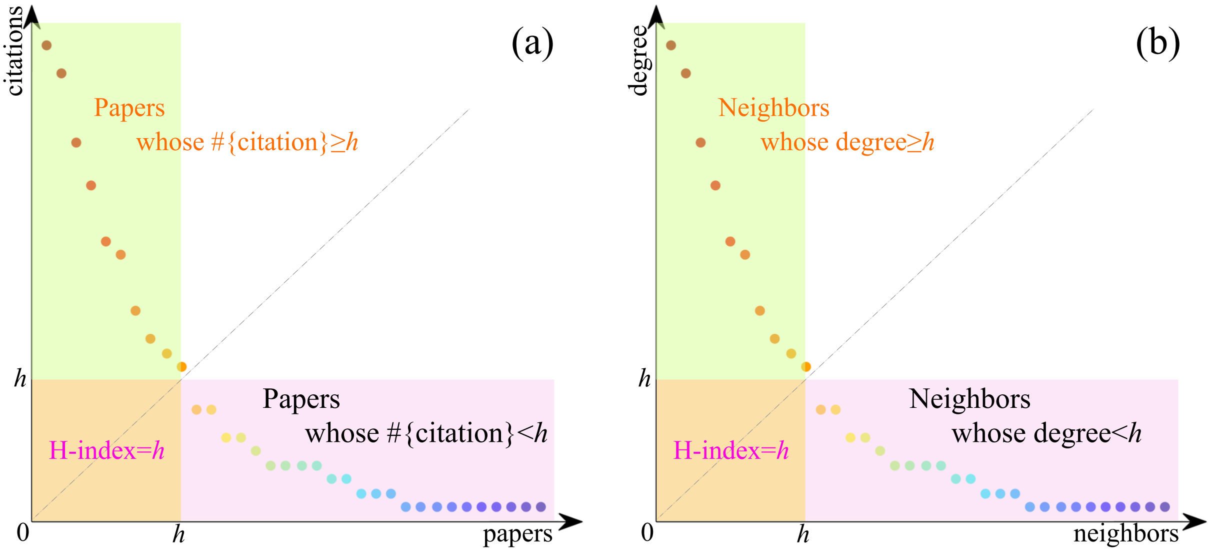


**Supplementary Figure 1. Explanation of calculating the H-index.** (a) the H-index of a scholar or a journal. In the 34 papers of this example we first rank all papers in decreasing order according to their citations, $n_i$, and pin them on the two-dimensional space according to the coordinate $(r_i, n_i)$, where $r_i$ is the ranking of paper $i$. We then locate the largest square without any points, but limited to the area between the axes and these points (orange area). The H-index will be equal to the side length of the square. (b) the H-index of a node in network. To calculate a node $j$'s H-index, the procedure is the same as the original one, but replacing the x-axis "papers" by its "neighbors" and the y-axis "citations" by its neighbors' "degree", i.e., $k_i$ where $i$ is the linked neighbor of node $j$. That is to say, we need to rank all $j$'s neighbors in decreasing order according to their degrees, $k_i$, and pin them on the two-dimensional space according to the coordinate $(r_i, k_i)$, where $r_i$ is the ranking of neighbor $i$. Then the H-index of node $j$ will equal to the side length of the largest square which does not contain any points and limited to the area between the axes and these points.

(a)

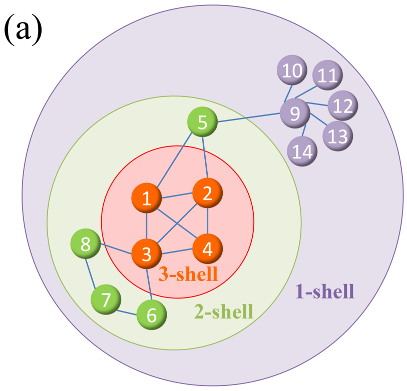


(b)

| Node | 1 | 2 | 3 | 4 | 5 | 6 | 7 | 8 | 9 | 10 | 11 | 12 | 13 | 14 |
|---|---|---|---|---|---|---|---|---|---|---|---|---|---|---|
| Degree | 4 | 4 | 5 | 3 | 3 | 2 | 2 | 2 | 6 | 1 | 1 | 1 | 1 | 1 |
| $h^{(1)}$-index | 3 | 3 | 3 | 3 | 3 | 2 | 2 | 2 | 1 | 1 | 1 | 1 | 1 | 1 |
| $h^{(2)}$-index (coreness) | 3 | 3 | 3 | 3 | 2 | 2 | 2 | 2 | 1 | 1 | 1 | 1 | 1 | 1 |
| $h^{(3)}$-index | 3 | 3 | 3 | 3 | 2 | 2 | 2 | 2 | 1 | 1 | 1 | 1 | 1 | 1 |

(c)

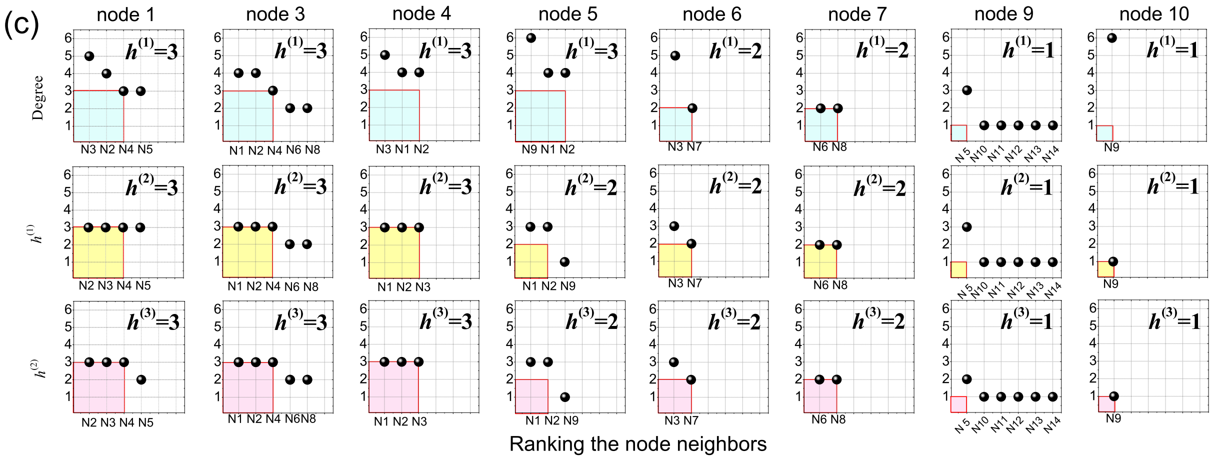


**Supplementary Figure 2. An example of iterative process from degree to coreness through calculating $h^{(n)}$-index.** (a) A simple network with 14 nodes and 18 edges. (b) The values of degree, $h^{(1)}$, $h^{(2)}$ and $h^{(3)}$ of the 14 nodes. (c) The procedure to calculating $h^{(n)}$ value for each node.

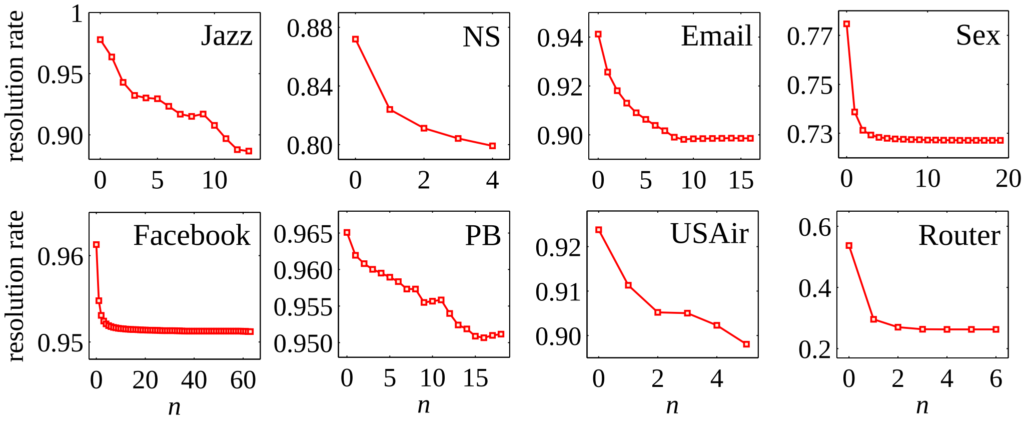


**Supplementary Figure 3. Resolution rate of $h^{(n)}$-index of the eight real-world undirected networks.** The value of $n$ ranges from 0 to $n_\infty$, corresponding to degree to coreness.

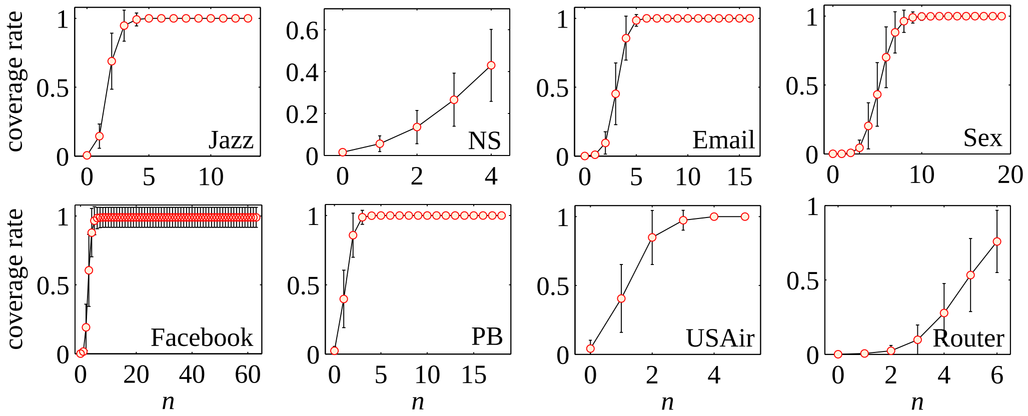


**Supplementary Figure 4. Coverage rate (with error bar) of $h^{(n)}$-index of eight real-world undirected networks.** The value of $n$ ranges from 0 to $n_{\infty}$, corresponding to degree to coreness..

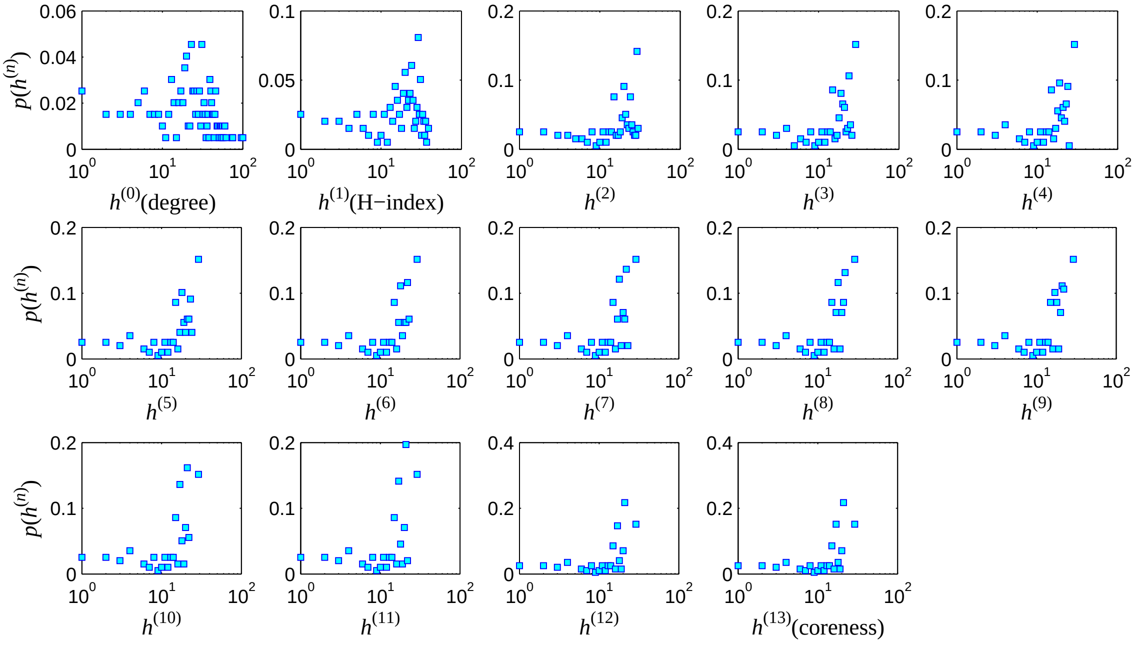


**Supplementary Figure 5. Distribution of $h^{(n)}$-index $(n = 0, 1, 2, \cdots, 13)$ of Jazz network.**

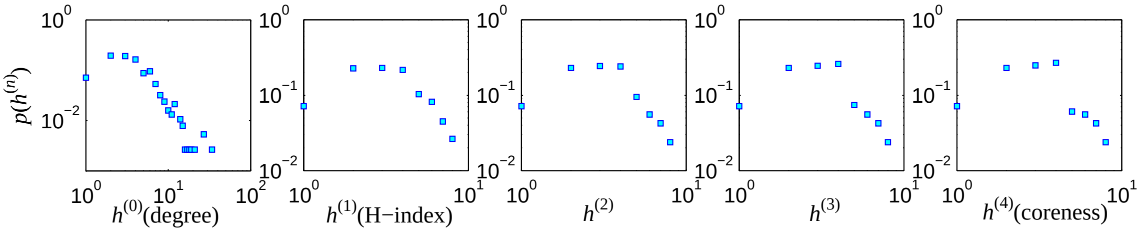


**Supplementary Figure 6. Distribution of $h^{(n)}$-index $(n = 0, 1, 2, 3, 4)$ of NS network.**

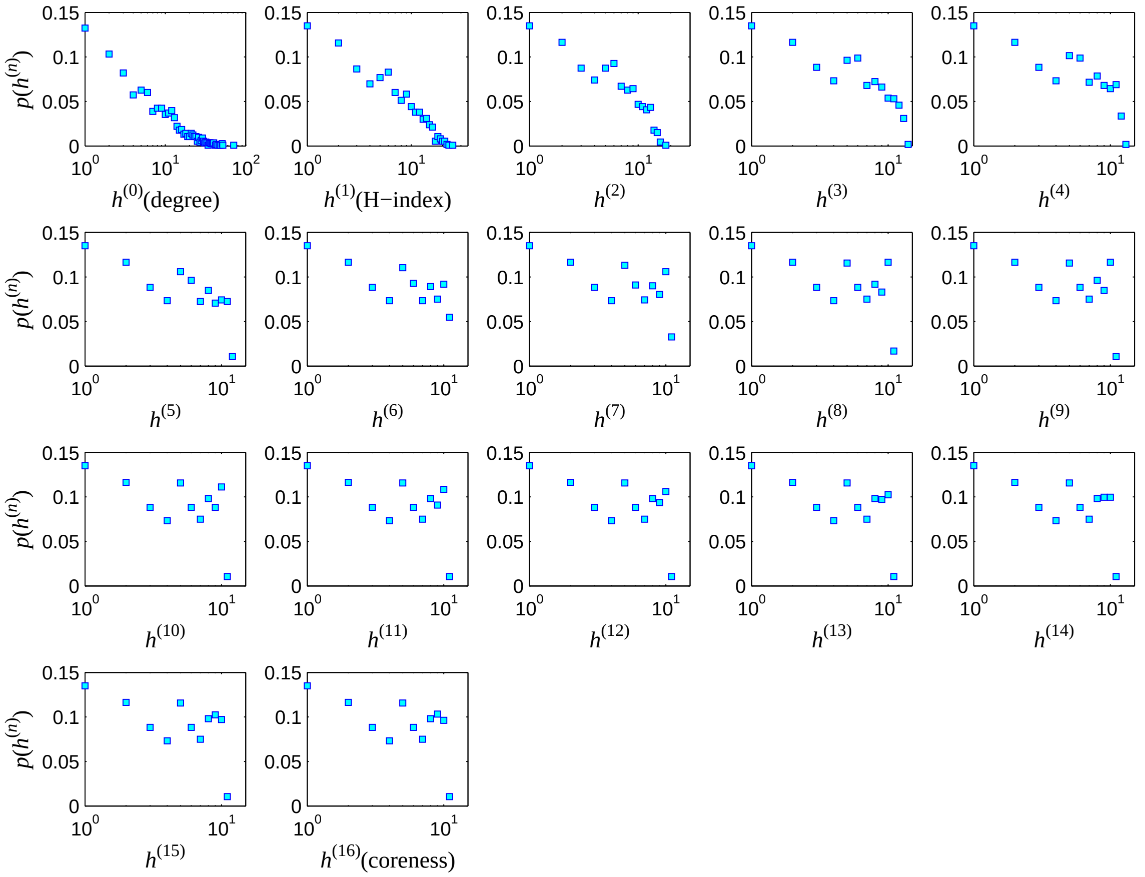


**Supplementary Figure 7. Distribution of $h^{(n)}$-index $(n = 0, 1, 2, \cdots, 16)$ of Email network.**

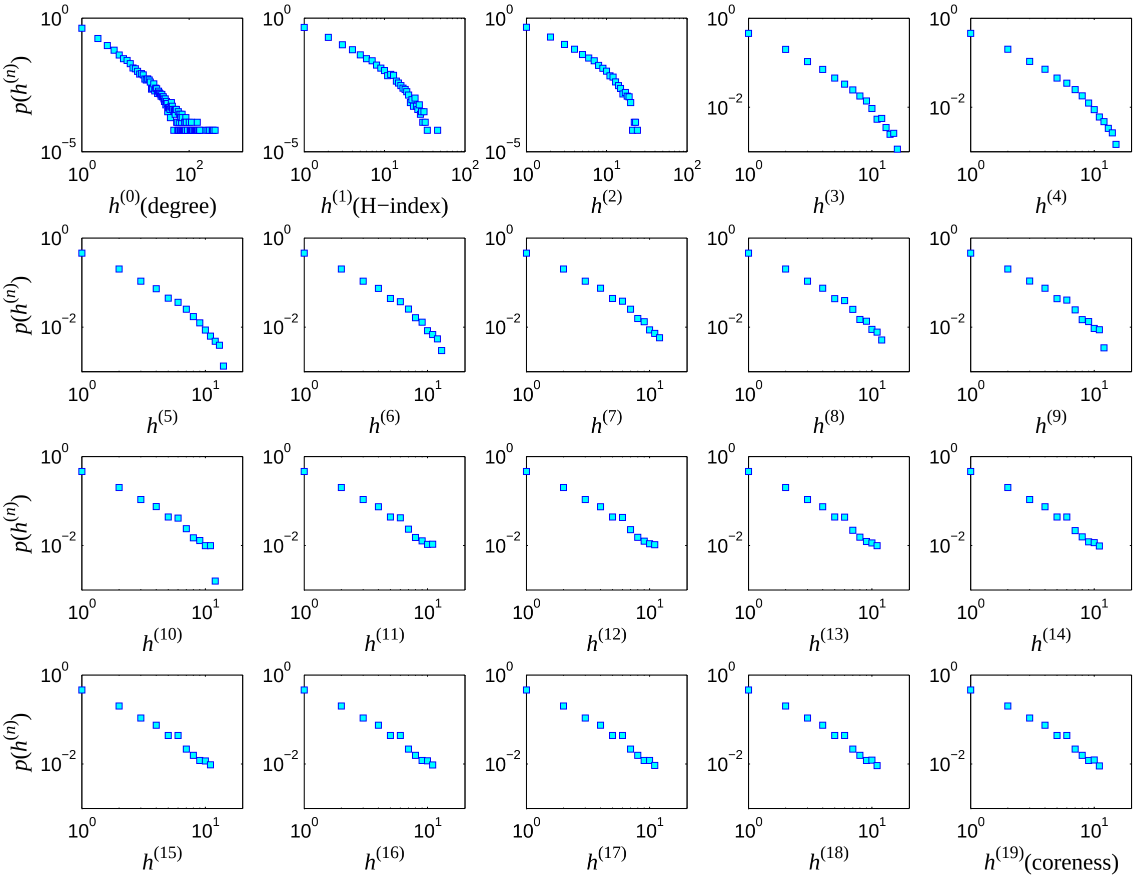


**Supplementary Figure 8. Distribution of $h^{(n)}$-index $(n = 0, 1, 2, \cdots, 19)$ of Sex network.**

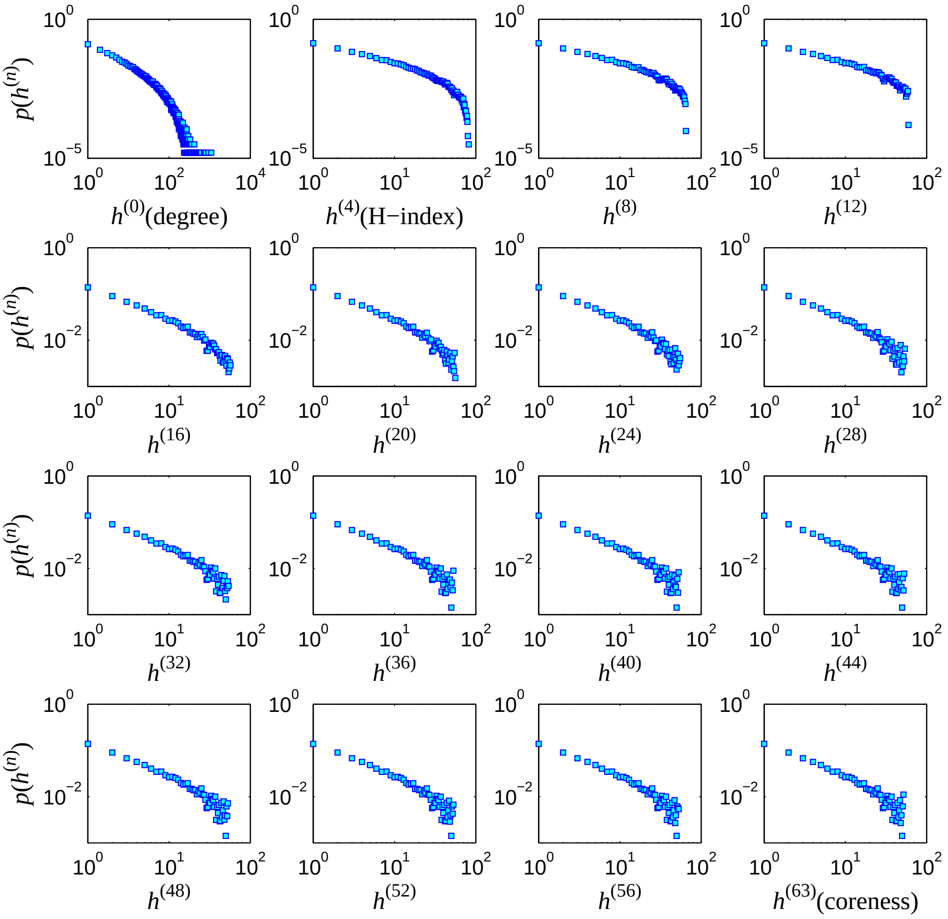


**Supplementary Figure 9. Distribution of $h^{(n)}$-index ($n = 0, 4, 8, 12 \cdots, 63$) of Facebook network.** We only show some representative examples.

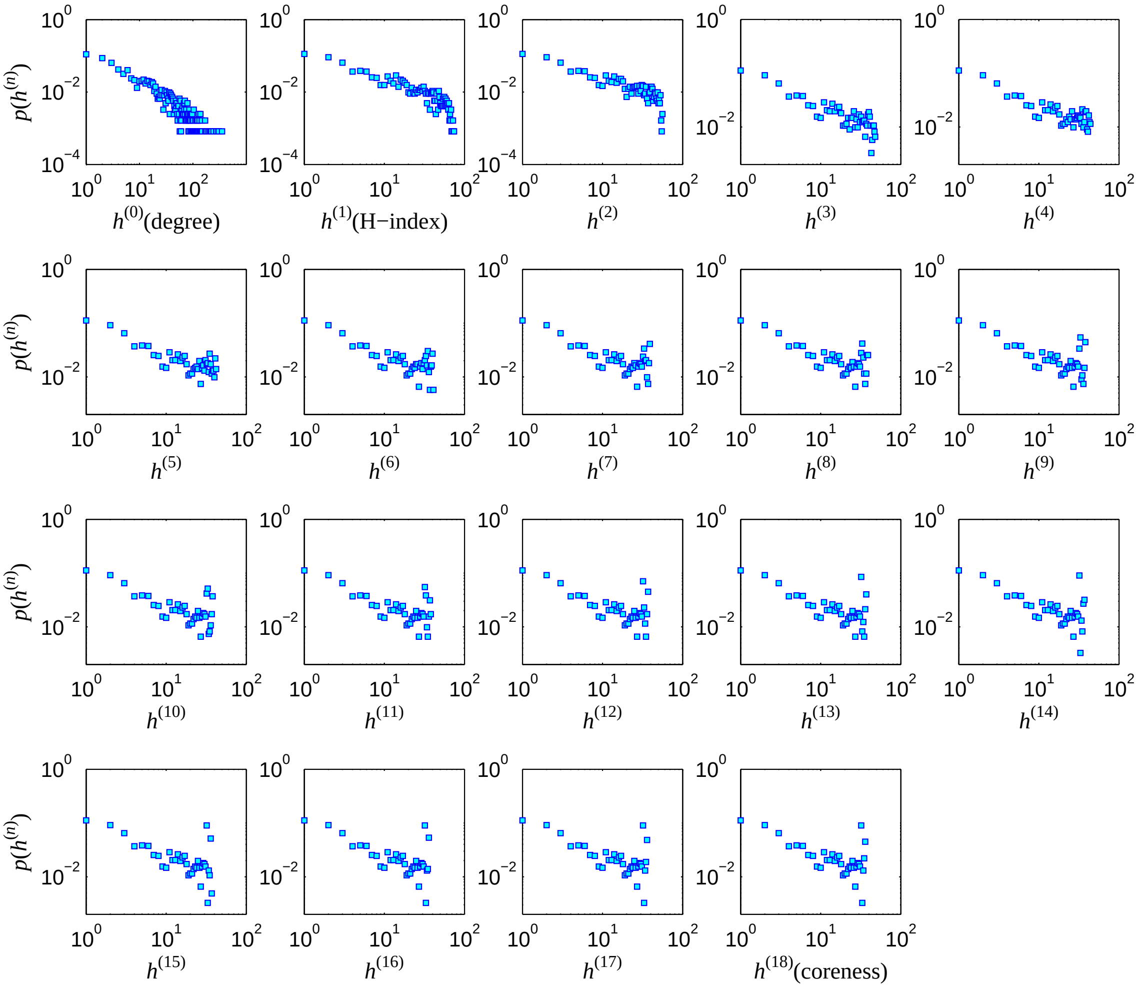


**Supplementary Figure 10. Distribution of $h^{(n)}$-index ($n = 0, 1, 2, \cdots, 18$) of PB network.**

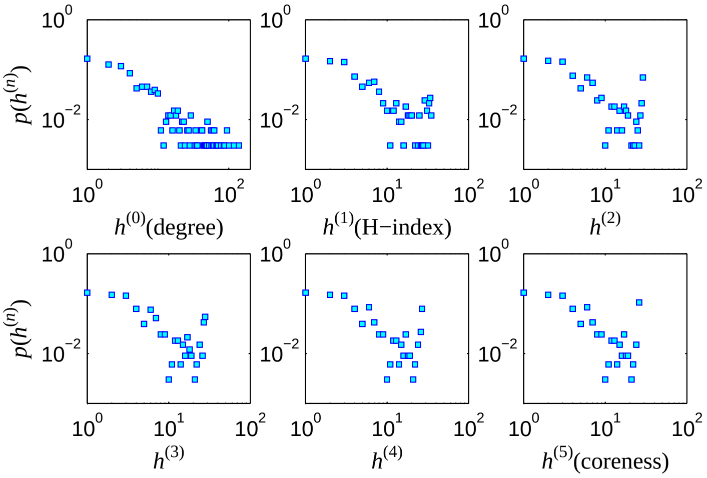


**Supplementary Figure 11. Distribution of $h^{(n)}$-index $(n = 0, 1, 2, 3, 4, 5)$ of USAir network.**

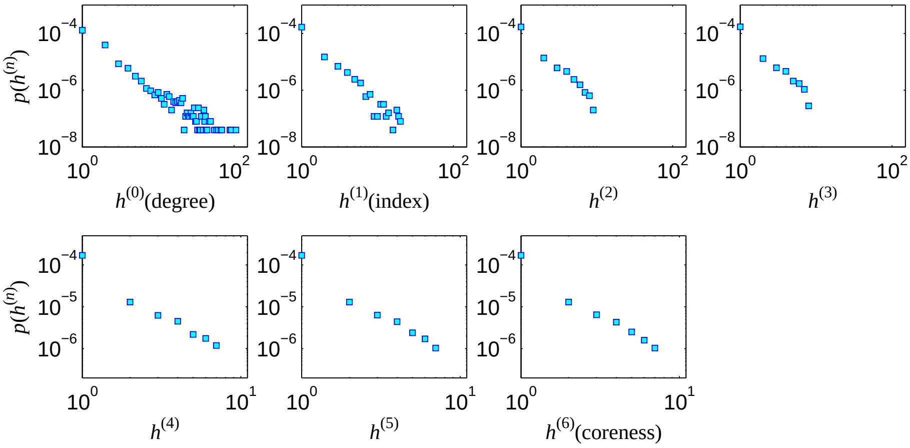


**Supplementary Figure 12. Distribution of $h^{(n)}$-index ($n = 0, 1, 2, 3, 4, 5, 6$) of Router network.**

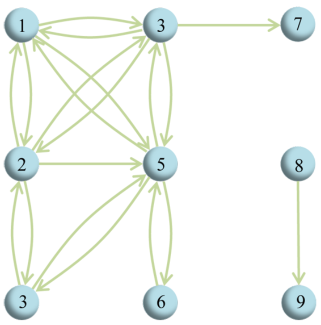


| Node | $h_{in}^{(0)}$ | $h_{in}^{(1)}$ | $h_{in}^{(2)}$ | $h_{in}^{(\infty)}$ | $h_{out}^{(0)}$ | $h_{out}^{(1)}$ | $h_{out}^{(2)}$ | $h_{out}^{(\infty)}$ |
|---|---|---|---|---|---|---|---|---|
| **1** | 3 | 3 | 2 | 2 | 3 | 3 | 2 | 2 |
| **2** | 3 | 2 | 2 | 2 | 4 | 3 | 2 | 2 |
| **3** | 2 | 2 | 2 | 2 | 2 | 2 | 2 | 2 |
| **4** | 3 | 3 | 2 | 2 | 4 | 3 | 2 | 2 |
| **5** | 5 | 3 | 2 | 2 | 4 | 2 | 2 | 2 |
| **6** | 1 | 1 | 1 | 1 | 1 | 1 | 1 | 1 |
| **7** | 1 | 1 | 1 | 1 | 0 | 0 | 0 | 0 |
| **8** | 0 | 0 | 0 | 0 | 1 | 0 | 0 | 0 |
| **9** | 1 | 0 | 0 | 0 | 0 | 0 | 0 | 0 |

**Supplementary Figure 13. The illustration of the calculation of $\mathrm{H_{in}}$-indices and $\mathrm{H_{out}}$-indices for a simple directed network.** In such example network containing nine nodes, the largest in-coreness and out-coreness are all 2.

# Supplementary Tables

**Supplementary Table 1. The Kendall Tau between the node influence index $R$ of SIR model and five centrality indices in undirected networks.** The indices are degree (i.e., $h^{(0)}$), H-index (i.e., $h^{(1)}$), coreness (i.e., $h^{(\infty)}$), closeness and betweenness. The spreading rate $\beta$ is set as $\beta = 2\beta_c$. In each row, the largest $\tau$ is highlighted in bold.

| Networks | degree | H-index | coreness | closeness | betweenness |
|---|---|---|---|---|---|
| Jazz | 0.8270 | **0.8616** | 0.7843 | 0.6975 | 0.4728 |
| NS | 0.4672 | **0.4788** | 0.4407 | 0.3948 | 0.3165 |
| Email | 0.8371 | **0.8643** | 0.8468 | 0.7744 | 0.6581 |
| Sex | 0.4601 | 0.5066 | 0.5230 | **0.7361** | 0.3865 |
| Facebook | 0.7318 | 0.7582 | **0.7709** | 0.7002 | 0.4875 |
| PB | 0.8640 | **0.8812** | 0.8733 | 0.7629 | 0.6823 |
| USAir | 0.7254 | 0.7552 | 0.7594 | **0.7737** | 0.5449 |
| Router | 0.3080 | 0.2811 | 0.2914 | **0.6233** | 0.3009 |

**Supplementary Table 2. The Kendall Tau between the node influence index $R$ of SIR model and five centrality indices in undirected networks.** The indices are degree (i.e., $h^{(0)}$), H-index (i.e., $h^{(1)}$), coreness (i.e., $h^{(\infty)}$), closeness and betweenness. The spreading rate $\beta$ is set as $\beta = 2.5\beta_c$. In each row, the largest $\tau$ is highlighted in bold.

| Networks | degree | H-index | coreness | closeness | betweenness |
|---|---|---|---|---|---|
| Jazz | 0.8601 | **0.8675** | 0.7710 | 0.7166 | 0.5034 |
| NS | 0.4577 | **0.4717** | 0.4370 | 0.4087 | 0.2929 |
| Email | 0.8528 | **0.8768** | 0.8593 | 0.7594 | 0.6639 |
| Sex | 0.5021 | 0.5544 | 0.5716 | **0.7250** | 0.4205 |
| Facebook | 0.7521 | 0.7818 | **0.7922** | 0.7216 | 0.4954 |
| PB | 0.8787 | **0.8910** | 0.8808 | 0.7642 | 0.6898 |
| USAir | 0.7554 | 0.7839 | **0.7876** | 0.7704 | 0.5613 |
| Router | 0.3232 | 0.3096 | 0.3244 | **0.6200** | 0.3121 |

**Supplementary Table 3. The Kendall Tau between the node influence index $R$ of SIS model and five centrality indices in undirected networks.** The indices are degree (i.e., $h^{(0)}$), H-index (i.e., $h^{(1)}$), coreness (i.e., $h^{(\infty)}$), closeness and betweenness. The spreading rate $\beta$ is set as $\beta = 1.5\beta_c$. In each row, the largest $\tau$ is highlighted in bold.

| Networks | degree | H-index | coreness | closeness | betweenness |
|---|---|---|---|---|---|
| Jazz | 0.7856 | **0.8134** | 0.7622 | 0.6652 | 0.4277 |
| NS | 0.5776 | **0.6040** | 0.5551 | 0.3113 | 0.3426 |
| Email | 0.7239 | **0.7505** | 0.7464 | 0.7317 | 0.5921 |
| Sex | 0.4254 | 0.4739 | 0.4923 | **0.6986** | 0.3570 |
| Facebook | 0.6310 | 0.6538 | **0.6662** | 0.6107 | 0.4230 |
| PB | 0.8404 | **0.8524** | 0.8467 | 0.7470 | 0.6832 |
| USAir | 0.7556 | 0.7844 | **0.7874** | 0.7482 | 0.5651 |
| Router | 0.3219 | 0.3049 | 0.3196 | **0.6040** | 0.3136 |

**Supplementary Table 4. The Kendall Tau between the node influence index $R$ of SIS model and five centrality indices in undirected networks.** The indices are degree (i.e., $h^{(0)}$), H-index (i.e., $h^{(1)}$), coreness (i.e., $h^{(\infty)}$), closeness and betweenness. The spreading rate $\beta$ is set as $\beta = 2\beta_c$. In each row, the largest $\tau$ is highlighted in bold.

| Networks | degree | H-index | coreness | closeness | betweenness |
|---|---|---|---|---|---|
| Jazz | 0.8071 | **0.8121** | 0.7369 | 0.6793 | 0.4709 |
| NS | 0.6822 | **0.7050** | 0.6585 | 0.2820 | 0.3790 |
| Email | 0.7842 | **0.8108** | 0.8015 | 0.7747 | 0.6290 |
| Sex | 0.4852 | 0.5390 | 0.5563 | **0.7216** | 0.4074 |
| Facebook | 0.6931 | 0.7205 | **0.7328** | 0.6761 | 0.4597 |
| PB | 0.8669 | **0.8780** | 0.8710 | 0.7640 | 0.6912 |
| USAir | 0.7669 | 0.7975 | **0.8005** | 0.7685 | 0.5701 |
| Router | 0.3528 | 0.3433 | 0.3601 | **0.5932** | 0.3371 |

**Supplementary Table 5. The Kendall Tau between the node influence index $R$ of SIS model and five centrality indices in undirected networks.** The indices are degree (i.e., $h^{(0)}$), H-index (i.e., $h^{(1)}$), coreness (i.e., $h^{(\infty)}$), closeness and betweenness. The spreading rate $\beta$ is set as $\beta = 2.5\beta_c$. In each row, the largest $\tau$ is highlighted in bold.

| Networks | degree | H-index | coreness | closeness | betweenness |
|---|---|---|---|---|---|
| Jazz | **0.8037** | 0.8006 | 0.7512 | 0.6765 | 0.4776 |
| NS | **0.7314** | 0.7311 | 0.6867 | 0.2581 | 0.4155 |
| Email | 0.8177 | **0.8391** | 0.8239 | 0.7595 | 0.6491 |
| Sex | 0.5318 | 0.5881 | 0.6041 | **0.7043** | 0.4470 |
| Facebook | 0.7330 | 0.7621 | **0.7724** | 0.7113 | 0.4831 |
| PB | 0.8685 | **0.8779** | 0.8710 | 0.7614 | 0.6916 |
| USAir | 0.7579 | 0.7852 | **0.7857** | 0.7533 | 0.5730 |
| Router | 0.3679 | 0.3604 | 0.3793 | **0.5742** | 0.3494 |

**Supplementary Table 6. The Kendall Tau between the node influence index $R$ of BP model and five centrality indices in undirected networks.** $R$ is measured by the probability of belonging to the giant connected component. The five centrality indices are degree (i.e., $h^{(0)}$), H-index (i.e., $h^{(1)}$), coreness (i.e., $h^{(\infty)}$), closeness and betweenness. The probability $p$ with which a link is occupied is set as $1.5p_c$. In each row, the largest $\tau$ is highlighted in bold.

| Networks | degree | H-index | coreness | closeness | betweenness |
|---|---|---|---|---|---|
| Jazz | 0.8554 | **0.8896** | 0.8189 | 0.7151 | 0.4768 |
| NS | 0.3447 | 0.3480 | 0.3188 | **0.4139** | 0.2607 |
| Email | 0.8370 | **0.8713** | 0.8553 | 0.8063 | 0.6552 |
| Sex | 0.4124 | 0.4642 | 0.4822 | **0.8397** | 0.3438 |
| Facebook | 0.7050 | 0.7362 | **0.7543** | 0.7456 | 0.4659 |
| PB | 0.9061 | **0.9265** | 0.9166 | 0.7965 | 0.6987 |
| USAir | 0.7588 | 0.7926 | 0.7942 | **0.8067** | 0.5636 |
| Router | 0.3627 | 0.3692 | 0.3921 | **0.5995** | 0.3413 |

**Supplementary Table 7. The Kendall Tau between the node influence index $R$ of BP model and five centrality indices in undirected networks.** $R$ is measured by the probability of belonging to the giant connected component. The five centrality indices are degree (i.e., $h^{(0)}$), H-index (i.e., $h^{(1)}$), coreness (i.e., $h^{(\infty)}$), closeness and betweenness. The probability $p$ with which a link is occupied is set as $2p_c$. In each row, the largest $\tau$ is highlighted in bold.

| Networks | degree | H-index | coreness | closeness | betweenness |
|---|---|---|---|---|---|
| Jazz | 0.9124 | **0.9186** | 0.8003 | 0.7313 | 0.5120 |
| NS | 0.3547 | 0.3635 | 0.3350 | **0.4397** | 0.2542 |
| Email | 0.8746 | **0.9045** | 0.8799 | 0.7860 | 0.6753 |
| Sex | 0.4767 | 0.5327 | 0.5512 | **0.8231** | 0.3974 |
| Facebook | 0.7285 | 0.7599 | **0.7727** | 0.7193 | 0.4786 |
| PB | 0.9246 | **0.9405** | 0.9282 | 0.7929 | 0.7088 |
| USAir | 0.7790 | 0.8123 | **0.8145** | 0.7947 | 0.5690 |
| Router | 0.3823 | 0.3976 | 0.4194 | **0.5507** | 0.3580 |

**Supplementary Table 8. The Kendall Tau between the node influence index $R$ of BP model and five centrality indices in undirected networks.** $R$ is measured by the probability of belonging to the giant connected component. The five centrality indices are degree (i.e., $h^{(0)}$), H-index (i.e., $h^{(1)}$), coreness (i.e., $h^{(\infty)}$), closeness and betweenness. The probability $p$ with which a link is occupied is set as $2.5p_c$. In each row, the largest $\tau$ is highlighted in bold.

| Networks | degree | H-index | coreness | closeness | betweenness |
|---|---|---|---|---|---|
| Jazz | **0.9346** | 0.9048 | 0.7790 | 0.7329 | 0.5287 |
| NS | 0.4064 | 0.4218 | 0.3928 | **0.4339** | 0.2652 |
| Email | 0.8986 | **0.9207** | 0.8900 | 0.7696 | 0.6867 |
| Sex | 0.5459 | 0.6059 | 0.6236 | **0.7620** | 0.4560 |
| Facebook | 0.7624 | 0.7954 | **0.8047** | 0.7434 | 0.4983 |
| PB | 0.9348 | **0.9463** | 0.9332 | 0.7881 | 0.7141 |
| USAir | 0.7917 | 0.8239 | **0.8251** | 0.7856 | 0.5793 |
| Router | 0.4001 | 0.4130 | 0.4374 | **0.5296** | 0.3731 |

**Supplementary Table 9. The Kendall Tau between the node influence index $R$ of BP model and five centrality indices in undirected networks.** $R$ is measured by the size of the connected component containing the node. The five centrality indices are degree (i.e., $h^{(0)}$), H-index (i.e., $h^{(1)}$), coreness (i.e., $h^{(\infty)}$), closeness and betweenness. The probability $p$ with which a link is occupied is set as $1.5p_c$. In each row, the largest $\tau$ is highlighted in bold.

| Networks | degree | H-index | coreness | closeness | betweenness |
|---|---|---|---|---|---|
| Jazz | 0.8688 | **0.8963** | 0.8137 | 0.7225 | 0.4861 |
| NS | 0.5184 | **0.5277** | 0.4870 | 0.3765 | 0.3406 |
| Email | 0.8388 | **0.8727** | 0.8560 | 0.8049 | 0.6563 |
| Sex | 0.4269 | 0.4762 | 0.4935 | **0.8361** | 0.3570 |
| Facebook | 0.7171 | 0.7459 | **0.7631** | 0.7444 | 0.4751 |
| PB | 0.9063 | **0.9262** | 0.9160 | 0.7959 | 0.6987 |
| USAir | 0.7668 | 0.7994 | 0.8006 | **0.8045** | 0.5690 |
| Router | 0.3653 | 0.3715 | 0.3941 | **0.5956** | 0.3435 |

**Supplementary Table 10. The Kendall Tau between the node influence index $R$ of BP model and five centrality indices in undirected networks.** $R$ is measured by the size of the connected component containing the node. The five centrality indices are degree (i.e., $h^{(0)}$), H-index (i.e., $h^{(1)}$), coreness (i.e., $h^{(\infty)}$), closeness and betweenness. The probability $p$ with which a link is occupied is set as $2p_c$. In each row, the largest $\tau$ is highlighted in bold.

| Networks | degree | H-index | coreness | closeness | betweenness |
|---|---|---|---|---|---|
| Jazz | 0.9153 | **0.9185** | 0.7973 | 0.7313 | 0.5139 |
| NS | 0.4801 | **0.4886** | 0.4508 | 0.4082 | 0.3213 |
| Email | 0.8751 | **0.9045** | 0.8797 | 0.7852 | 0.6756 |
| Sex | 0.4809 | 0.5362 | 0.5544 | **0.8172** | 0.4013 |
| Facebook | 0.7434 | 0.7716 | **0.7832** | 0.7121 | 0.4924 |
| PB | 0.9244 | **0.9401** | 0.9278 | 0.7922 | 0.7087 |
| USAir | 0.7831 | 0.8156 | **0.8172** | 0.7925 | 0.5714 |
| Router | 0.3841 | 0.3983 | 0.4196 | **0.5502** | 0.3597 |

**Supplementary Table 11. The Kendall Tau between the node influence index $R$ of BP model and five centrality indices in undirected networks.** $R$ is measured by the size of the connected component containing the node. The five centrality indices are degree (i.e., $h^{(0)}$), H-index (i.e., $h^{(1)}$), coreness (i.e., $h^{(\infty)}$), closeness and betweenness. The probability $p$ with which a link is occupied is set as $2.5p_c$. In each row, the largest $\tau$ is highlighted in bold.

| Networks | degree | H-index | coreness | closeness | betweenness |
|---|---|---|---|---|---|
| Jazz | **0.9345** | 0.9030 | 0.7766 | 0.7316 | 0.5292 |
| NS | 0.4872 | **0.5051** | 0.4693 | 0.4021 | 0.3046 |
| Email | 0.8986 | **0.9203** | 0.8894 | 0.7690 | 0.6866 |
| Sex | 0.5474 | 0.6071 | 0.6246 | **0.7579** | 0.4574 |
| Facebook | 0.7645 | 0.7950 | **0.8035** | 0.7315 | 0.5020 |
| PB | 0.9346 | **0.9458** | 0.9327 | 0.7875 | 0.7140 |
| USAir | 0.7954 | 0.8266 | **0.8272** | 0.7850 | 0.5820 |
| Router | 0.4015 | 0.4133 | 0.4371 | **0.5293** | 0.3745 |

**Supplementary Table 12. The basic topological features and the converging time of the seven directed networks.** $|V|$ and $|E|$ are the number of nodes and links, respectively. $\langle k \rangle$ is the average degree. $\langle d \rangle$ is the average distance taking into account only the connected node pairs. $C$ is the clustering coefficient for directed networks [14]. $P_{\text{weak}}$ is the proportion of nodes belonging to the largest weakly connected component, and $P_{\text{strong}}$ is the proportion of nodes belonging to the largest strongly connected component. $n_\infty$ is the converging time to in-coreness, defined as the minimum steps required to reach in-coreness from in-degree by the operator $\mathcal{H}$.

| Networks | $\|V\|$ | $\|E\|$ | $\langle k \rangle$ | $\langle d \rangle$ | $C$ | $P_{\text{weak}}$ | $P_{\text{strong}}$ | $n_\infty$ |
|---|---|---|---|---|---|---|---|---|
| PB(D) | 1222 | 19021 | 15.565 | 3.39 | 0.21 | 1 | 0.6489 | 17 |
| P2P | 8114 | 26013 | 3.206 | 7.055 | 0.005 | 0.9988 | 0.3234 | 13 |
| Epinions | 75879 | 508837 | 6.706 | 5.96 | 0.106 | 1 | 0.4247 | 41 |
| Anybeat | 12645 | 67053 | 5.303 | 3.286 | 0.172 | 1 | 0.6736 | 18 |
| ASCAIDA | 26475 | 106762 | 4.033 | 3.876 | 0.208 | 1 | 1 | 12 |
| FBLike | 1899 | 20296 | 10.688 | 3.197 | 0.085 | 0.9968 | 0.6814 | 30 |
| WikiVote | 7115 | 103689 | 14.573 | 3.341 | 0.081 | 0.9931 | 0.1827 | 22 |

**Supplementary Table 13. The Kendall Tau between the node influence index $R$ of SIR model and five centrality indices in directed networks.** The indices are in-degree (i.e., $h_{\mathrm{in}}^{(0)}$), in-H-index (i.e., $h_{\mathrm{in}}^{(1)}$), in-coreness (i.e., $h_{\mathrm{in}}^{(\infty)}$), PageRank and Authority. The spreading rate $\beta$ is set as $\beta = 1.5\beta_c$. In each row, the largest $\tau$ is highlighted in bold.

| Networks | in-degree | in-H-index | in-coreness | PageRank | Authority |
|---|---|---|---|---|---|
| PB(D) | 0.3324 | 0.3489 | **0.3512** | 0.2909 | 0.3324 |
| P2P | **0.1496** | 0.1319 | 0.0017 | 0.1081 | **0.1496** |
| Epinions | 0.2612 | 0.2670 | **0.2689** | 0.2307 | 0.2612 |
| Anybeat | 0.2670 | 0.2836 | 0.2861 | **0.3543** | 0.2670 |
| ASCAIDA | 0.4372 | 0.4677 | **0.4730** | 0.2483 | 0.4372 |
| FBLike | **0.6845** | 0.6844 | 0.6790 | 0.6541 | **0.6845** |
| WikiVote | -0.0140 | 0.0278 | **0.0286** | -0.0191 | -0.0140 |

**Supplementary Table 14. The Kendall Tau between the node influence index $R$ of SIR model and five centrality indices in directed networks.** The indices are in-degree (i.e., $h_{\text{in}}^{(0)}$), in-H-index (i.e., $h_{\text{in}}^{(1)}$), in-coreness (i.e., $h_{\text{in}}^{(\infty)}$), PageRank and Authority. The spreading rate $\beta$ is set as $\beta = 2\beta_c$. In each row, the largest $\tau$ is highlighted in bold.

| Networks | in-degree | in-H-index | in-coreness | PageRank | Authority |
|---|---|---|---|---|---|
| PB(D) | 0.3290 | 0.3457 | **0.3482** | 0.2897 | 0.3290 |
| P2P | **0.1502** | 0.1316 | 0.0038 | 0.1090 | **0.1502** |
| Epinions | 0.2496 | 0.2555 | **0.2577** | 0.2188 | 0.2496 |
| Anybeat | 0.2748 | 0.2913 | 0.2938 | **0.3557** | 0.2748 |
| ASCAIDA | 0.4562 | 0.4896 | **0.4950** | 0.2542 | 0.4562 |
| FBLike | 0.6895 | **0.6901** | 0.6846 | 0.6600 | 0.6895 |
| WikiVote | -0.0123 | 0.0297 | **0.0305** | -0.0174 | -0.0123 |

**Supplementary Table 15. The Kendall Tau between the node influence index $R$ of SIR model and five centrality indices in directed networks.** The indices are in-degree (i.e., $h_{\text{in}}^{(0)}$), in-H-index (i.e., $h_{\text{in}}^{(1)}$), in-coreness (i.e., $h_{\text{in}}^{(\infty)}$), PageRank and Authority. The spreading rate $\beta$ is set as $\beta = 2.5\beta_c$. In each row, the largest $\tau$ is highlighted in bold.

| Networks | in-degree | in-H-index | in-coreness | PageRank | Authority |
|---|---|---|---|---|---|
| PB(D) | 0.3274 | 0.3435 | **0.3454** | 0.2878 | 0.3274 |
| P2P | **0.1509** | 0.1333 | 0.0032 | 0.1093 | **0.1509** |
| Epinions | 0.2443 | 0.2509 | **0.2532** | 0.2124 | 0.2443 |
| Anybeat | 0.2812 | 0.2979 | 0.3006 | **0.3513** | 0.2812 |
| ASCAIDA | 0.4783 | 0.5137 | **0.5197** | 0.2635 | 0.4783 |
| FBLike | **0.6925** | 0.6921 | 0.6866 | 0.6643 | **0.6925** |
| WikiVote | -0.0106 | 0.0313 | **0.0322** | -0.0158 | -0.0106 |

**Supplementary Table 16. The Kendall Tau between the node influence index $R$ of SIS model and five centrality indices in directed networks.** The indices are in-degree (i.e., $h_{\text{in}}^{(0)}$), in-H-index (i.e., $h_{\text{in}}^{(1)}$), in-coreness (i.e., $h_{\text{in}}^{(\infty)}$), PageRank and Authority. The spreading rate $\beta$ is set as $\beta = 1.5\beta_c$. In each row, the largest $\tau$ is highlighted in bold.

| Networks | in-degree | in-H-index | in-coreness | PageRank | Authority |
|---|---|---|---|---|---|
| PB(D) | 0.8358 | **0.8701** | 0.8651 | 0.7929 | 0.8358 |
| P2P | 0.6010 | **0.6589** | 0.2448 | 0.6301 | 0.6010 |
| Epinions | 0.4035 | 0.4179 | **0.4209** | 0.3616 | 0.4035 |
| Anybeat | 0.4691 | 0.5020 | **0.5069** | 0.4211 | 0.4691 |
| ASCAIDA | 0.4073 | 0.4392 | **0.4443** | 0.2308 | 0.4073 |
| FBLike | 0.7826 | **0.7924** | 0.7877 | 0.6715 | 0.7826 |
| WikiVote | 0.6629 | 0.6971 | **0.7006** | 0.6204 | 0.6629 |

**Supplementary Table 17. The Kendall Tau between the node influence index $R$ of SIS model and five centrality indices in directed networks.** The indices are in-degree (i.e., $h_{\text{in}}^{(0)}$), in-H-index (i.e., $h_{\text{in}}^{(1)}$), in-coreness (i.e., $h_{\text{in}}^{(\infty)}$), PageRank and Authority. The spreading rate $\beta$ is set as $\beta = 2\beta_c$. In each row, the largest $\tau$ is highlighted in bold.

| Networks | in-degree | in-H-index | in-coreness | PageRank | Authority |
|---|---|---|---|---|---|
| PB(D) | 0.8591 | **0.8893** | 0.8809 | 0.8104 | 0.8591 |
| P2P | 0.6532 | **0.7062** | 0.2567 | 0.6656 | 0.6532 |
| Epinions | 0.4896 | 0.5099 | **0.5133** | 0.4245 | 0.4896 |
| Anybeat | 0.5254 | 0.5616 | **0.5668** | 0.4345 | 0.5254 |
| ASCAIDA | 0.4345 | 0.4685 | **0.4740** | 0.2410 | 0.4345 |
| FBLike | 0.8327 | **0.8397** | 0.8350 | 0.7156 | 0.8327 |
| WikiVote | 0.7270 | 0.7828 | **0.7870** | 0.6868 | 0.727 |

**Supplementary Table 18. The Kendall Tau between the node influence index $R$ of SIS model and five centrality indices in directed networks.** The indices are in-degree (i.e., $h_{\text{in}}^{(0)}$), in-H-index (i.e., $h_{\text{in}}^{(1)}$), in-coreness (i.e., $h_{\text{in}}^{(\infty)}$), PageRank and Authority. The spreading rate $\beta$ is set as $\beta = 2.5\beta_c$. In each row, the largest $\tau$ is highlighted in bold.

| Networks | in-degree | in-H-index | in-coreness | PageRank | Authority |
|---|---|---|---|---|---|
| PB(D) | 0.8685 | **0.8984** | 0.8894 | 0.8051 | 0.8685 |
| P2P | 0.6869 | **0.7271** | 0.2600 | 0.6855 | 0.6869 |
| Epinions | 0.5482 | 0.5730 | **0.5770** | 0.4656 | 0.5482 |
| Anybeat | 0.5641 | 0.6019 | **0.6075** | 0.4482 | 0.5641 |
| ASCAIDA | 0.4591 | 0.4949 | **0.5009** | 0.2552 | 0.4591 |
| FBLike | 0.8537 | **0.8644** | 0.8604 | 0.7269 | 0.8537 |
| WikiVote | 0.7550 | 0.8232 | **0.8277** | 0.7095 | 0.7550 |

**Supplementary Table 19. The Kendall Tau between the node influence index $R$ of BP model and five centrality indices in directed networks.** $R$ is measured by the probability of belonging to the giant strongly connected component. The five indices are in-degree (i.e., $h_{\text{in}}^{(0)}$), in-H-index (i.e., $h_{\text{in}}^{(1)}$), in-coreness (i.e., $h_{\text{in}}^{(\infty)}$), PageRank and Authority. The probability $p$ with which a link is occupied is set as $1.5p_c$. In each row, the largest $\tau$ is highlighted in bold.

| Networks | in-degree | in-H-index | in-coreness | PageRank | Authority |
|---|---|---|---|---|---|
| PB(D) | 0.6804 | 0.7047 | **0.7057** | 0.6275 | 0.6804 |
| P2P | **0.2716** | 0.2691 | 0.1294 | 0.2279 | **0.2716** |
| Epinions | 0.4910 | 0.5072 | **0.5102** | 0.4276 | 0.4910 |
| Anybeat | 0.5386 | 0.5668 | **0.5702** | 0.5051 | 0.5386 |
| ASCAIDA | 0.4038 | 0.4328 | **0.4372** | 0.2796 | 0.4038 |
| FBLike | 0.7813 | **0.7833** | 0.7735 | 0.7152 | 0.7813 |
| WikiVote | 0.4431 | 0.4503 | **0.4513** | 0.4023 | 0.4431 |

**Supplementary Table 20. The Kendall Tau between the node influence index $R$ of BP model and five centrality indices in directed networks.** $R$ is measured by the probability of belonging to the giant strongly connected component. The five indices are in-degree (i.e., $h_{\text{in}}^{(0)}$), in-H-index (i.e., $h_{\text{in}}^{(1)}$), in-coreness (i.e., $h_{\text{in}}^{(\infty)}$), PageRank and Authority. The probability $p$ with which a link is occupied is set as $2p_c$. In each row, the largest $\tau$ is highlighted in bold.

| Networks | in-degree | in-H-index | in-coreness | PageRank | Authority |
|---|---|---|---|---|---|
| PB(D) | 0.6929 | 0.7161 | **0.7171** | 0.6298 | 0.6929 |
| P2P | **0.2650** | 0.2591 | 0.1295 | 0.2156 | **0.2650** |
| Epinions | 0.5433 | 0.5619 | **0.5651** | 0.4641 | 0.5433 |
| Anybeat | 0.5390 | 0.5669 | **0.5704** | 0.5168 | 0.5390 |
| ASCAIDA | 0.4644 | 0.4965 | **0.5023** | 0.3109 | 0.4644 |
| FBLike | 0.8032 | **0.8046** | 0.7953 | 0.7378 | 0.8032 |
| WikiVote | 0.4495 | **0.4566** | **0.4566** | 0.4100 | 0.4495 |

**Supplementary Table 21. The Kendall Tau between the node influence index $R$ of BP model and five centrality indices in directed networks.** $R$ is measured by the probability of belonging to the giant strongly connected component. The five indices are in-degree (i.e., $h_{\text{in}}^{(0)}$), in-H-index (i.e., $h_{\text{in}}^{(1)}$), in-coreness (i.e., $h_{\text{in}}^{(\infty)}$), PageRank and Authority. The probability $p$ with which a link is occupied is set as $2.5p_c$. In each row, the largest $\tau$ is highlighted in bold.

| Networks | in-degree | in-H-index | in-coreness | PageRank | Authority |
|---|---|---|---|---|---|
| PB(D) | 0.6919 | 0.7153 | **0.7165** | 0.6268 | 0.6919 |
| P2P | **0.2660** | 0.2560 | 0.1288 | 0.2139 | **0.2660** |
| Epinions | 0.5762 | 0.5970 | **0.6001** | 0.4867 | 0.5762 |
| Anybeat | 0.5461 | 0.5738 | **0.5772** | 0.5267 | 0.5461 |
| ASCAIDA | 0.5037 | 0.5402 | **0.5460** | 0.3273 | 0.5037 |
| FBLike | **0.8010** | **0.8010** | 0.7917 | 0.7418 | **0.8010** |
| WikiVote | 0.4492 | 0.4557 | **0.4560** | 0.4100 | 0.4492 |

**Supplementary Table 22. The Kendall Tau between the node influence index $R$ of BP model and five centrality indices in directed networks.** $R$ is measured by the size of the strongly connected component containing the node. The five indices are in-degree (i.e., $h_{\mathrm{in}}^{(0)}$), in-H-index (i.e., $h_{\mathrm{in}}^{(1)}$), in-coreness (i.e., $h_{\mathrm{in}}^{(\infty)}$), PageRank and Authority. The probability $p$ with which a link is occupied is set as $1.5p_c$. In each row, the largest $\tau$ is highlighted in bold.

| Networks | in-degree | in-H-index | in-coreness | PageRank | Authority |
|---|---|---|---|---|---|
| PB(D) | 0.6777 | 0.7012 | **0.7018** | 0.6262 | 0.6777 |
| P2P | **0.2709** | 0.2685 | 0.1292 | 0.2272 | **0.2709** |
| Epinions | 0.5243 | 0.5394 | **0.5416** | 0.4647 | 0.5243 |
| Anybeat | 0.5303 | 0.5547 | **0.5571** | 0.5284 | 0.5303 |
| ASCAIDA | 0.4240 | 0.4356 | **0.4366** | 0.3235 | 0.4240 |
| FBLike | 0.7762 | **0.7779** | 0.7688 | 0.7199 | 0.7762 |
| WikiVote | 0.4427 | 0.4504 | **0.4512** | 0.4026 | 0.4427 |

**Supplementary Table 23. The Kendall Tau between the node influence index $R$ of BP model and five centrality indices in directed networks.** $R$ is measured by the size of the strongly connected component containing the node. The five indices are in-degree (i.e., $h_{\text{in}}^{(0)}$), in-H-index (i.e., $h_{\text{in}}^{(1)}$), in-coreness (i.e., $h_{\text{in}}^{(\infty)}$), PageRank and Authority. The probability $p$ with which a link is occupied is set as $2p_c$. In each row, the largest $\tau$ is highlighted in bold.

| Networks | in-degree | in-H-index | in-coreness | PageRank | Authority |
|---|---|---|---|---|---|
| PB(D) | 0.6894 | 0.7128 | **0.7140** | 0.6285 | 0.6894 |
| P2P | **0.2647** | 0.2588 | 0.1294 | 0.2154 | **0.2647** |
| Epinions | 0.5758 | 0.5929 | **0.5949** | 0.5043 | 0.5758 |
| Anybeat | 0.5334 | 0.5586 | **0.5614** | 0.5353 | 0.5334 |
| ASCAIDA | 0.4733 | 0.4906 | **0.4939** | 0.3499 | 0.4733 |
| FBLike | 0.7959 | **0.7968** | 0.7875 | 0.7413 | 0.7959 |
| WikiVote | 0.4483 | **0.4556** | 0.4555 | 0.4093 | 0.4483 |

**Supplementary Table 24. The Kendall Tau between the node influence index $R$ of BP model and five centrality indices in directed networks.** $R$ is measured by the size of the strongly connected component containing the node. The five indices are in-degree (i.e., $h_{\mathrm{in}}^{(0)}$), in-H-index (i.e., $h_{\mathrm{in}}^{(1)}$), in-coreness (i.e., $h_{\mathrm{in}}^{(\infty)}$), PageRank and Authority. The probability $p$ with which a link is occupied is set as $2.5p_c$. In each row, the largest $\tau$ is highlighted in bold.

| Networks | in-degree | in-H-index | in-coreness | PageRank | Authority |
|---|---|---|---|---|---|
| PB(D) | 0.6905 | 0.7132 | **0.7146** | 0.6282 | 0.6905 |
| P2P | **0.2656** | 0.2554 | 0.1286 | 0.2135 | **0.2656** |
| Epinions | 0.6095 | 0.6285 | **0.6308** | 0.5336 | 0.6095 |
| Anybeat | 0.5426 | 0.5678 | **0.5706** | 0.5398 | 0.5426 |
| ASCAIDA | 0.5048 | 0.5302 | **0.5343** | 0.3591 | 0.5048 |
| FBLike | **0.7938** | 0.7927 | 0.7836 | 0.7438 | **0.7938** |
| WikiVote | 0.4488 | 0.4553 | **0.4555** | 0.4097 | 0.4488 |

**Supplementary Table 25.** Notations used in the paper.

| Notations | Description |
|---|---|
| $N$ | The number of nodes in the given network |
| $V$ | The set of nodes in the given network |
| $E$ | The set of edges in the given network |
| $\mathcal{H}$ | The operator $\mathcal{H}$ |
| $k_i$ | The degree of node $i$ |
| $\langle k \rangle$ | The average degree in the given network |
| $\langle d \rangle$ | The average distance in the given network |
| $C$ | The clustering coefficient of the given network |
| $r$ | The assortative coefficient of the given network |
| $c_i$ | The coreness of node $i$ |
| $h_i^{(0)}$ | The zero-order H-index of node $i$, $h_i^{(0)} = k_i$ |
| $h_i^{(n)}$ | The $n$-order H-index of node $i$ |
| $h_i$ | The H-index of node $i$, i.e., the first-order H-index, $h_i = h_i^{(1)}$ |
| $n_\infty$ | The convergence time |
| $h_i^{(n_\infty)}$ | The steady state, $h_i^{(n_\infty)} = c_i$ |
| $\beta$ | The probability that a susceptible node will be infected by one of its infected neighbors in the SIR model |
| $\lambda$ | The probability that a infected node will be removed in the SIR model |
| $\lambda_{\mathrm{SIS}}$ | The probability that a infected node will become to susceptible node in the SIS model |
| $p$ | The probability with which a link is occupied in bond percolation. |
| $R_i$ | The influence of node $i$, which is the average number of removed nodes after the dynamics over 1000 independent runs, each of which begins with node $i$ as the sole infected seed |
| $\tau$ | The Kendall Tau coefficient |
| $F^{(n)}$ | The $n$-order resolution rate |
| $C_i^{(n)}$ | The $n$-order coverage rate of node $i$ |

# Supplementary Note 1: The definition of $h^{(n)}$-index in complex networks

## a. Definition of H-index (i.e., $h^{(1)}$) in complex networks

Degree, H-index, and coreness are well-known indices:

**(i)** Degree: the number of neighbors linked to one node $i$, denoted $k_i$.

**(ii)** H-index [1]: a scholar's H-index is the maximum value $h$ such that there exist at least $h$ papers, each with a citation count $\geq h$; see Fig. 1(a) for the method of calculating the H-index.

**(iii)** Coreness [2]: a node is of coreness $k$ if it belongs to the $k$-core but not to the $(k+1)$-core. The $k$-core of a graph is the maximal subgraph in which each node has at least degree $k$ in the subgraph.

The H-index was originally used to measure the citation impact of a scholar or a journal. We now extend this concept to networks. We assume node $i$ to be a scholar, its neighbors to be the papers by $i$, and the degree of each neighbor to be the citations to each paper (i.e., each neighbor). We thus define the H-index ($h_i$) of node $i$ (i.e., $h_i^{(1)}$) to be the largest value such that node $i$ has at least $h_i$ neighbors of degree no less than $h_i$. In Supplementary Fig. 1, we compare the original H-index (subfigure (a)) with the H-index in complex networks (subfigure (b)).

## b. Extending to high-order indices ($n > 2$)

Using the degree of the neighbors, we define the H-index. Then using this H-index, we similarly define the $h^{(2)}$ index, the second-order H-index. Iteratively, we can extend the H-index to higher orders $h^{(n)}$. We define the initial state $h^{(0)} = k$ and the first-order H-index $h^{(1)}$. The iterative calculation of $h_i^{(n)}$ for node $i$ is:

$h_i^{(1)}$: $h_i^{(1)} = h_i = h$, if $i$ has $h$ neighbors whose degrees (i.e., $h^{(0)}$) are at least $h$, the other $(k_i - h)$ neighbors' degrees are less than $h$.

$h_i^{(2)}$: $h_i^{(2)} = h$, if $i$ has $h$ neighbors whose $h^{(1)}$ are at least $h$, the other $(k_i - h)$ neighbors' $h^{(1)}$ are less than $h$.

...

$h_i^{(n)}$: $h_i^{(n)} = h$, if $i$ has $h$ neighbors whose $h^{(n-1)}$ are at least $h$, the other $(k_i - h)$ neighbors' $h^{(n-1)}$ are less than $h$.

We mathematically construct an operator $\mathcal{H}$ that acts on a finite number of reals $(x_1, x_2, \cdots, x_n)$ and returns an integer $y = \mathcal{H}(x_1, x_2, \cdots, x_n) > 0$, where $y$ is the maximum integer such that there exist at least $y$ elements in $(x_1, x_2, \cdots, x_n)$, each of which is no less than $y$. Denote $G(V, E)$ an undirected simple network, where $V$ is the set of nodes and $E$ is the set of links. The degree of an arbitrary node $i$ is denoted by $k_i$ and its neighbors' degrees are $k_{j_1}, k_{j_2}, \cdots, k_{j_{k_i}}$. We then define $h_i^{(0)} = k_i$ to be the zero-order H-index of node $i$, and define the $n$-order H-index $(n > 0)$ iteratively as

$$h_i^{(n)} = \mathcal{H}\left(h_{j_1}^{(n-1)}, h_{j_2}^{(n-1)}, \cdots, h_{j_{k_i}}^{(n-1)}\right). \tag{1}$$

The H-index of a node is equal to the first-order H-index, i.e., $h_i^{(1)}$.

## c. An example of how to calculate $h^{(n)}$-index in synchronous manner

Supplementary Fig. 2 shows an example of how to calculate $h^{(n)}$ in a synchronous manner. Take node 1 as the example. We first rank its four neighbors in decreasing order according to their degree $k$ (i.e., $h^{(0)}$ values), and pin them on the two-dimensional space according to the coordinate $(r_i, h_i^{(0)})$, where $r_i$ is the ranking of its neighbor $i$, as shown in the first two-dimensional space in subfigure (c). We then determine the largest square that contains no nodes, but limit our search to the area between the axes and these nodes. In the case of node 1, the largest square is $3 \times 3$, therefore $h_1^{(1)} = 3$. In this example, the iteration converges in two steps. The $h^{(2)}$ values are then the exact coreness values of the nodes.

## d. Definition of H-family indices in directed networks

We extend the H-family indices (defined above) from undirected to directed networks, where degree is replaced by in-degree or out-degree during the iteration process. Supplementary Fig. 13 shows a simple example of calculating $\mathrm{H_{in}}$-indices and $\mathrm{H_{out}}$-indices. Accordingly, $h_{\mathrm{in/out}}^{(0)}$, $h_{\mathrm{in/out}}^{(1)}$ and $h_{\mathrm{in/out}}^{(\infty)}$ correspond to in/out-degree, in/out-H-index and in/out-coreness, respectively.

## Supplementary Note 2: Resolution rate of $h^{(n)}$-index

The resolution rate of the $h^{(n)}$-index is the probability that two randomly chosen nodes will have a different $h^{(n)}$. It is also a useful index for measuring the degree to which a network is coarse grained. Mathematically it can be obtained from the distribution of $h^{(n)}$ (i.e., $p(h^{(n)})$),

$$F^{(n)} = 1 - \sum_{h^{(n)}} P(h^{(n)})^2, \tag{2}$$

where the summation runs over all distinct values of $h^{(n)}$. Supplementary Fig. 3 shows the resolution rate of the eight real networks. The results show that the higher the order of $h^{(n)}$, the lower the resolution rate. Degree has the highest resolution rate and coreness the lowest.

## Supplementary Note 3: Coverage rate of $h^{(n)}$-index

For any node $i$ and $n$-order H-index, the information coverage is defined as the ratio between the number of nodes with distance no more than $n$ from $i$ and the size of the network, which quantifies the information required to calculate $h_i^{(n)}$. The information coverage of the whole network is the average value of all nodes, which increases as $n$ increases.

To calculate the coverage rate, we assume that the degree of each node in the network is known. For the $h^{(0)}$-index (i.e., degree) we only use the information of the node itself, and thus the coverage of one node is $1/N$ where $N$ is the number of nodes in the network. For the $h^{(1)}$-index we must know the degree information of the node's neighbors, then the coverage of one node $j$ is $|\Gamma_j|/N$, where $\Gamma_j$ is the set of the neighbors of node $i$. For the $h^{(2)}$-index we must know the $h^{(1)}$-index information of the node's neighbors, and to obtain the $h^{(1)}$-index of the node's neighbors we must know the degree of the neighbors of the node's neighbors, i.e., we require node information that can be reached in two steps. We denote the set of nodes that node $j$ can reach in $n$ steps ($n = 2, \cdots, n_\infty$) to be $\Gamma_j^{(n)}$. We then define the $h^{(n)}$-coverage of one node $j$ to be

$$C_j^{(n)} = \frac{1}{N}|j \cup \Gamma_j \cup \Gamma_j^{(2)} \cdots \cup \Gamma_j^{(n)}|. \tag{3}$$

Averaging over all nodes, we obtain the coverage rate of the network,

$$C^{(n)} = \frac{1}{N}\sum_{j=1}^{N} C_j^{(n)}. \tag{4}$$

Note that $C^{(0)} = 1/N$ and $C^{(1)} = 2|E|/N^2 + 1/N$. Supplementary Fig. 4 shows the coverage rates of the $h^{(n)}$-indices of the eight real-world networks. In some cases, e.g., NS and Router, the information coverage surprisingly is lower than 1 even for $h^{(n_\infty)}$. This indicates that calculating the coreness of a node in these networks requires only partial information. Specifically, to calculate the coreness of node $i$ whose convergence time is $n_\infty = t$, we only need to focus on the subgraph that contains node $i$, the nodes with distance no greater than $t+1$ from $i$, and all links between them. The nodes with distnce $t+1$ from $i$ are the boundary nodes. Although the updating process is essentially the same as before, the $h^{(n)}$ values of all boundary nodes must not be changed during the updating process, i.e., the $h^{(n)}$ values of these nodes are always equal to their degree.

## Supplementary Note 4: Kendall Tau between the node influence and the centrality indices for undirected networks

In the main text we show the Kendall Tau between the node influence $R$ for the SIR model (with spreading rate $1.5\beta_c$) in undirected networks and the five centrality indices, including the representative H-family indices (i.e., degree, H-index, and coreness) and two well-known centrality indices (i.e., closeness and betweenness). To verify, we conduct additional experiments using different spreading rates, and we introduce two other dynamical processes, i.e., the susceptible-infected-susceptible (SIS) model [3], and the bond percolation (BP) model [4]. The corresponding experiments and results are presented below.

### a. Comparisons of Kendall Tau for SIR model in undirected networks

In Supplementary Table 1 and 2, the spreading rate $\beta$ of the SIR model is set at $2\beta_c$ and $2.5\beta_c$ respectively. The optimal index for each network is consistent with the results when $\beta = 1.5\beta_c$ in the main body.

### b. Comparisons of Kendall Tau for SIS model in undirected networks

In contrast to the SIR model, infected individuals in the susceptible-infected-susceptible (SIS) model [3] do not recover and with a probability $\lambda_{\text{SIS}}$ again become susceptible. We initialize the SIS process by infecting 20% of the individuals. The number of infected individuals eventually reaches a dynamic equilibrium state in which as many infectious individuals become susceptible as susceptible nodes become infectious. Note that the epidemic continues only when the spreading rate $\beta$ is larger than the threshold $\beta_c$. To quantify $\beta_c$ in these networks, we set $\lambda_{\text{SIS}} = 0.1$ and implement a large number of experiments to simulate the SIS process with $\beta$ values ranging from 0 to 1. This gives us the threshold $\beta_c$ values for Jazz, NS, Email, Sex, Facebook, PB, USAir, and Router, which are approximately 0.0036, 0.022, 0.005, 0.0041, 0.0008, 0.0016, 0.0039, and 0.01, respectively.

The node influence $R$ in the SIS model is defined as the probability that a node will be infected in the steady state. In our experiments, the values of $R$ are obtained using 200 independent implementations. We present the Kendall Tau between the node influence index $R$ and the five centrality indices in Supplementary Table 3, 4 and 5, where the spreading rate $\beta$ values are set to $1.5\beta_c$, $2\beta_c$, and $2.5\beta_c$, respectively.

## c. Comparisons of Kendall Tau for BP model in undirected networks

In a connected network, bond percolation (BP) [4] is process in which every link (or bond) is independently removed with a fixed probability $1 - p$. Each link is thus occupied with a probability $p$ and otherwise vacant. Note that as $p$ approaches 1 a cluster (a group of nodes connected by occupied links) percolates through the network. As we decrease $p$ the probability of a cluster forming also decreases. In our experiment we set the $p$ values to be $1.5p_c$, $2p_c$, and $2.5p_c$ respectively, where $p_c$ is the threshold at which a cluster percolating through the network emerges. To quantity $p_c$, we run 100 independent experiments with $p$ values ranging from 0 to 1, check the largest cluster, and find approximate $p_c$ values for Jazz, NS, Email, Sex, Facebook, PB, USAir, and Router of 0.033, 0.15, 0.067, 0.042, 0.009, 0.017, 0.032, and 0.2, respectively.

We use the BP model to measure node influence $R$, which is defined in two ways: (i) the probability that the target node belongs to the giant component (i.e., cluster) and (ii) the size of the connected component containing the target node. We run 1000 independent implementations for a fixed $p$ to obtain these two types of $R$ value. We first present the Kendall Tau between the node influence index $R$ (measured using the probability that the node belongs to the giant connected component) and the five centrality indices in Supplementary Table 6, 7, and 8, where the probabilities $p$ are set at $1.5p_c$, $2p_c$, and $2.5p_c$, respectively. We then show the results for the other case in Supplementary Table 9, 10, and 11, where the probabilities $p$ are set at $1.5p_c$, $2p_c$, and $2.5p_c$, respectively.

# Supplementary Note 5: Kendall Tau between the node influence and the centrality indices for directed networks

For directed networks, we choose PageRank [5] and Authority (one of the two indices in HITs [6]) to form comparisons with the representative H-family indices (i.e., in-degree, $H_{in}$-index and in-coreness).

## a. Statistical features of directed networks

We select seven directed networks for our comparison. (i) PB(D) [7], the directed version of the PB network, recording connections between the US political blogs. (ii) P2P [8], a snapshot of the Gnutella peer-to-peer file sharing network from August 2002. (iii) Epinions [9], a who-trust-whom online social network of a general consumer review site Epinions.com. Members of the site can decide whether to "trust" each other. (iv) Anybeat [10], an online community from a public gathering place where you can interact with people from your neighborhood or across the world. (v) ASCAIDA [11], containing 122 CAIDA AS graphs, from January 2004 to November 2007. (vi) FBLike [12], a Facebook-like social network originating from an online community for students at the University of California, Irvine. The dataset includes users who have sent or received at least one message. (vii) WikiVote [13], the record of votes promoting someone to administratorship in Wikipedia. Nodes represent Wikipedia users and a directed link from node $i$ to $j$ indicates that user $i$ voted to promote user $j$. The network contains all the voting data from the inception of Wikipedia until January 2008. The basic statistics of these seven directed networks are summarized in Supplementary Table 12.

## b. Comparisons of Kendall Tau for SIR model in directed networks

In directed networks the spread of a disease follows the directions of the links. For example, an infected node $u$ is able to infect a susceptible node $v$ if the directed link $\langle u, v \rangle$ exists. Similar to the SIR process in undirected networks, the influence $R$ of node $u$ is quantified using the average number of removed nodes after 1000 independent runs, each of which begins with node $u$ as the sole infected seed. We then use simulation experiments to also estimate the thresholds and find approximate $\beta_c$ values for the seven directed networks (i.e., PB(D), P2P, Epinions, Anybeat, ASCAIDA, FBLike and WikiVote) to be 0.035, 0.025, 0.011, 0.019, 0.022, 0.031, and 0.025, respectively. We present the Kendall Tau between the node influence index $R$ and the five centrality indices in Supplementary Table 13, 14, and 15, where the

spreading rate $\beta$ values are set at $1.5\beta_c$, $2\beta_c$, and $2.5\beta_c$, respectively.

## c. Comparisons of Kendall Tau for SIS model in directed networks

As described above, in directed networks the spread of a disease follows the directions of the links. The SIS process in directed networks is the same as in undirected networks. Thus we use the SIS process to measure the influence $R$ of a node, defined as the probability that the node remains infected in the steady state, and we use simulations to estimate the threshold $\beta_c$. We find the approximate $\beta_c$ values for the seven directed networks (i.e., PB(D), P2P, Epinions, Anybeat, ASCAIDA, FBLike and WikiVote) to be 0.005, 0.03, 0.0009, 0.0018, 0.002, 0.003, and 0.002, respectively. We then present the Kendall Tau between the node influence index $R$ and the five centrality indices in Supplementary Table 16, 17, and 18, where the spreading rate $\beta$ values are set at $1.5\beta_c$, $2\beta_c$, and $2.5\beta_c$, respectively.

## d. Comparisons of Kendall Tau for BP model in directed networks

The BP process in directed networks is approximately the same as in undirected networks. The only difference is due to the definition of connected components in directed networks. If we use the weakly connected components, these networks would be treated as undirected networks. Thus we use the strongly connected components to measure the node influence index $R$. Similarly, we use simulation experiments to estimate the approximate $p_c$ values for the seven directed networks (i.e., PB(D), P2P, Epinions, Anybeat, ASCAIDA, FBLike and WikiVote), which are 0.05, 0.29, 0.02, 0.1, 0.03, 0.04 and 0.03, respectively.

We also use the BP model to determine values for the two kinds of node influence $R$ measurement. Supplementary Table 19, 20, and 21 present the Kendall Tau values between the node influence index $R$ (measured by the probability of belonging to the giant strongly connected component) and the five centrality indices. And Supplementary Table 22, 23, and 24 show the results of the Kendall Tau between the node influence index $R$ (measured by the size of the strongly connected component containing the node) and the five centrality indices.

# Supplementary References


[1] Hirsch, J. E. An index to quantify an individual's scientific research output. *Proceedings of the National Academy of Sciences of the United States of America* **102**, 16569–16572 (2005).

[2] Dorogovtsev, S. N., Goltsev, A. V. & Mendes, J. F. F. K-core organization of complex networks. *Physical Review Letters* **96**, 040601 (2006).

[3] Pastor-Satorras R., Castellano C., Van Mieghem P., & Vespignani A. Epidemic processes in complex networks. arXiv: 1408.2701, (2014).

[4] Broadbent, S. R. & Hammersley, J. M. Percolation processes I. Crystals and mazes. *Proceedings of the Cambridge Philosophical Society* **53**, 629C641 (1957).

[5] Brin S., & Page L. The Anatomy of a Large-Scale Hypertextual Web Search Engine. *Computer Networks and ISDN Systems* **30**, 107–117 (1998).

[6] Kleinberg J. Authoritative sources in a hyperlinked environment. *Journal of the ACM* **46**, 604–632 (1999).

[7] Adamic, L. A. & Glance, N. The political blogosphere and the 2004 US election: divided they bolg. in *Proceedings 3rd International Workshop on Link Discovery* (ACM, New York), 36–43 (2004).

[8] Leskovec, J., Kleinberg J. & Faloutsos, C. Graph Evolution: Densification and Shrinking Diameters. *ACM Transactions on Knowledge Discovery from Data* **1**, (2007).

[9] Richardson M., Agrawal R. & Domingos P. Trust Management for the Semantic Web. in *Proceedings 2nd International Semantic Web Conference*, 351–368 (2003).

[10] Fire, M., Puzis, R. & Elovici, Y. Link Prediction in Highly Fractional Data Sets. *Handbook of Computational Approaches to Counterterrorism* (2012).

[11] Leskovec J., Kleinberg J. & Faloutsos C. Graphs over Time: Densification Laws, Shrinking Diameters and Possible Explanations. in *ACM SIGKDD International Conference on Knowledge Discovery and Data Mining (KDD)*, (2005).

[12] Opsahl, T. & Panzarasa, P. Clustering in weighted networks. *Social Networks* **31**, 155–163 (2009).

[13] Leskovec J., Huttenlocher D. & Kleinberg J. Predicting Positive and Negative Links in Online Social Networks. in *Proceedings of the 19th International Conference on World Wide Web*, 641–650 (2010).

[14] Bastian M., Heymann S. & Jacomy M. Gephi: an open source software for exploring and manipulating networks. in *International AAAI Conference on Weblogs and Social Media*, (2009).
